# DNA methylation classifier to diagnose pancreatic ductal adenocarcinoma metastases from different anatomical sites

**DOI:** 10.1186/s13148-024-01768-x

**Published:** 2024-11-10

**Authors:** Teodor G. Calina, Eilís Perez, Elena Grafenhorst, Jamal Benhamida, Simon Schallenberg, Adrian Popescu, Ines Koch, Tobias Janik, BaoQing Chen, Jana Ihlow, Stephanie Roessler, Benjamin Goeppert, Bruno Sinn, Marcus Bahra, George A. Calin, Eliane T. Taube, Uwe Pelzer, Christopher C. M. Neumann, David Horst, Erik Knutsen, David Capper, Mihnea P. Dragomir

**Affiliations:** 1 TGC Ventures UG, Berlin, Germany; 2 Faculty of Physics, Babeș-Bolyai University, Cluj-Napoca, Romania; 3 Department of Neuropathology, Charité – Universitätsmedizin Berlin, Corporate Member of Freie Universität Berlin, Humboldt-Universität zu Berlin, Berlin, Germany; 4 Berlin School of Integrative Oncology (BSIO), Charite – Universitätsmedizin Berlin (CVK), Berlin, Germany; 5 Institute of Pathology, Charité – Universitätsmedizin Berlin, Corporate Member of Freie Universität Berlin, Humboldt-Universität zu Berlin, Charitéplatz 1, 10117 Berlin, Germany; 6 Department of Pathology and Laboratory Medicine, Memorial Sloan Kettering Cancer Center, New York, NY USA; 7 German Cancer Consortium (DKTK), Partner Site Berlin, and German Cancer Research Center (DKFZ), Heidelberg, Germany; 8 State Key Laboratory of Oncology in South China, Department of Radiation Oncology, Collaborative Innovation Center of Cancer Medicine, Sun Yat-sen University Cancer Center, Guangzhou, Guangdong People’s Republic of China; 9 Guangdong Esophageal Cancer Research Institute, Guangzhou, Guangdong People’s Republic of China; 10 Berlin Institute of Health, Berlin, Germany; 11 Institute of Pathology, Heidelberg University Hospital, Heidelberg, Germany; 12 Institute of Pathology and Neuropathology, Hospital RKH Kliniken Ludwigsburg, Ludwigsburg, Germany; 13 Institute of Tissue Medicine and Pathology (ITMP), University Bern, Bern, Switzerland; 14 Department of Surgical Oncology and Robotics, Krankenhaus Waldfriede, Lehrkrankenhaus der Charité, Berlin, Germany; 15 Department of Translational Molecular Pathology, The University of Texas MD Anderson Cancer Center, Houston, TX USA; 16 The RNA Interference and Non-Coding RNA Center, The University of Texas MD Anderson Cancer Center, Houston, TX USA; 17 Department of Hematology, Oncology and Tumor Immunology, Charité – Universitätsmedizin Berlin, Corporate Member of Freie Universität Berlin, Humboldt-Universität zu Berlin, Berlin, Germany; 18 Department of Medical Biology, Faculty of Health Sciences, UiT The Artic University of Norway, Tromsø, Norway

**Keywords:** Pancreatic ductal adenocarcinoma, DNA methylation, Molecular diagnosis, Cancer of unknown primary, Epigenetics

## Abstract

**Background:**

We have recently constructed a DNA methylation classifier that can discriminate between pancreatic ductal adenocarcinoma (PAAD) liver metastasis and intrahepatic cholangiocarcinoma (iCCA) with high accuracy (*PAAD-iCCA-Classifier*). PAAD is one of the leading causes of cancer of unknown primary and diagnosis is based on exclusion of other malignancies. Therefore, our focus was to investigate whether the *PAAD-iCCA-Classifier* can be used to diagnose PAAD metastases from other sites.

**Methods:**

For this scope, the anomaly detection filter of the initial classifier was expanded by 8 additional mimicker carcinomas, amounting to a total of 10 carcinomas in the negative class. We validated the updated version of the classifier on a validation set, which consisted of a biological cohort (*n* = 3579) and a technical one (*n* = 15). We then assessed the performance of the classifier on a test set, which included a positive control cohort of 16 PAAD metastases from various sites and a cohort of 124 negative control samples consisting of 96 breast cancer metastases from 18 anatomical sites and 28 carcinoma metastases to the brain.

**Results:**

The updated *PAAD-iCCA-Classifier* achieved 98.21% accuracy on the biological validation samples, and on the technical validation ones it reached 100%. The classifier also correctly identified 15/16 (93.75%) metastases of the positive control as PAAD, and on the negative control, it correctly classified 122/124 samples (98.39%) for a 97.85% overall accuracy on the test set. We used this DNA methylation dataset to explore the organotropism of PAAD metastases and observed that PAAD liver metastases are distinct from PAAD peritoneal carcinomatosis and primary PAAD, and are characterized by specific copy number alterations and hypomethylation of enhancers involved in epithelial-mesenchymal-transition.

**Conclusions:**

The updated *PAAD-iCCA-Classifier* (available at https://classifier.tgc-research.de/) can accurately classify PAAD samples from various metastatic sites and it can serve as a diagnostic aid.

**Supplementary Information:**

The online version contains supplementary material available at 10.1186/s13148-024-01768-x.

## Background

We have recently developed a DNA methylation classifier that can solve a difficult surgical pathology problem: differentiating between primary intrahepatic cholangiocarcinoma (iCCA) and liver metastases of pancreatic ductal adenocarcinoma (PAAD)–*PAAD-iCCA-Classifier* [1]. The classification pipeline starts by separating colon and gastric adenocarcinoma from PAAD, iCCA, and normal bile samples and excludes these from the final classification process. Samples advancing past the first layer are labeled as either PAAD, iCCA, or normal bile tissue, receiving a probability score for each class. The third layer filters out samples with a low probability score (below 0.8 for carcinomas and 0.5 for normal bile). Samples clearing all three layers are classified as either PAAD, iCCA or normal bile, while those excluded at any stage are assigned a "No Match" class.

Most cancers of unknown primary (CUP) are adenocarcinomas [2], and in large autopsy series, the second most common identified origin of CUPs is PAAD (24%) [3]. No current immunohistochemical and molecular pathological diagnostic tool can confirm this diagnosis. PAAD are immunohistochemically characterized by pan-cytokeratin (CK) positivity and more specifically they may be CK7 and CK20 positive or CK7 positive and CK20 negative [4]. A complete loss of SMAD4 is helpful in the diagnostic process, but this alteration is present in 55% of PAAD and is not specific for this entity [5]. In recent years, several tools have been tested to predict the tissue of origin of CUPs [4], and only a 2000-gene microarray-based expression assay, which has not been confirmed for PAAD, has been approved by the FDA [6]. In addition, targeted DNA sequencing is limited and most PAADs are characterized by nonspecific driver mutations, such as *KRAS*, *TP53* and *SMAD4* [7]. Due to the small size of the primary PAAD tumors, imaging may be of limited value in identifying the primary site [8]. Finally, several studies reported that a correct diagnosis of the primary tumor leads to improved management and prognosis [9, 10].

In recent years, DNA methylation has been proposed as a powerful tool for predicting the tissue of origin [11, 12]. We believe that such classifiers need to be built, trained and validated on large datasets. We used 467 PAAD samples from several geographic regions to develop our classifier and showed that for PAAD liver metastases, we can correctly diagnose 94.28% of tumors, achieving superior results compared to immunohistochemistry-based classifiers [1]. Therefore, the aim of this paper was to upgrade our *PAAD-iCCA-Classifier* to work in a new scenario—the diagnosis of PAAD metastases from other metastatic sites. We also used this dataset to gain biological insight into the epigenetic program of metastasis by comparing differentially methylated probes (DMP) between the metastatic sites and the primary PAAD tumors.

## Materials and methods

### Patient sets and study design

We used the same reference samples as in our previous publication, containing 205 primary PAAD, 144 primary iCCA, and 50 normal bile duct samples from 7 different studies [1].

For developing the anomaly detection layer, we put together the anomaly detection training samples, which included 20% of the samples from 10 different carcinomas from TCGA. These 10 carcinomas formed the negative class and were selected because of their high metastatic potential and their prevalence as possible differential diagnoses of PAAD in a metastatic setting. Briefly, we included: breast invasive carcinoma (BRCA, *n* = 159), esophageal carcinoma (ESCA, *n* = 35), lung adenocarcinoma (LUAD, *n* = 97), stomach adenocarcinoma (STAD, *n* = 79), liver hepatocellular carcinoma (LIHC, *n* = 78), colon adenocarcinoma (COAD, *n* = 63), rectum adenocarcinoma (READ, *n* = 21), uterine corpus endometrial carcinoma (UCEC, *n* = 93), cervical squamous cell carcinoma and endocervical adenocarcinoma (CESC, *n* = 62), and prostate adenocarcinoma (PRAD, *n* = 100). The reference samples were added to the anomaly detection layer to represent the positive class.

The biological validation cohort (*n* = 3579) consisted of 252 primary PAAD, 151 primary iCCA, and 20 normal bile duct samples partially representing the validation set for the initial classifier development [1], to which we added 16 primary PAAD samples, 20 PAAD liver metastases (PAAD met.^Liv.^) and 36 primary iCCAs (GSE217384) that represented our previous testing cohort, 26 previously published formalin fixed paraffin embedded (FFPE) primary PAAD from Benhamida et al. [13] and the additional 80% of the samples from the 10 mimicker carcinomas (*n* = 3120).

The technical validation cohort consisted of 6 not otherwise specified lung cancers and 5 BRCA samples that were analyzed using the Illumina Infinium MethylationEPIC v2.0 BeadChip (EPICv2) (Illumina, CA, USA) array (GEO GSE222919), as well as 4 *in-house* PAAD metastases: peritoneal carcinomatosis (PAAD met.^PC^), lymph node metastasis (PAAD met.^LN^), spleen metastasis (PAAD met.^Spleen^) and PAAD met.^Liv.^.

The performance of the classifier was tested on a test set. As positive control we used 16 confirmed or clinically highly suspected PAAD metastases: 11 PAAD met.^PC^, 2 PAAD met.^LN^, 2 PAAD lung metastases (PAAD met.^Lung^), and 1 PAAD met.^Liv.^.

The negative control, used to test the classifier’s performance in excluding potential mimickers, contained:

(i) a unique set of 96 BRCA metastasis from 18 different anatomical sites (adrenal gland *n* = 3, bone *n* = 3, brain *n* = 12, chest *n* = 7, gastrointestinal tract (GI tract) *n* = 2, kidney *n* = 1, liver *n* = 24, lung *n* = 11, lymph node *n* = 12, ovary *n* = 3, pancreas *n* = 1, pericardium and pleura *n* = 3, peritoneum *n* = 2, skin *n* = 2, soft tissue *n* = 7, spleen *n* = 1, thyroid *n* = 1, uterus *n* = 1) from the AURORA US network resource. This set contained BRCA of all different molecular subtypes, samples were both FFPE and fresh frozen and were obtained both from autopsies and pathology specimens. Methylation was performed using the Illumina Infinium MethylationEPIC BeadChip (EPICv1) (Illumina, CA, USA) array [14].
(ii) An external set of 13 carcinoma brain metastases (GSE249157): 1 BRCA, 7 LUAD, 4 COAD and 1 PRAD. All samples were fresh frozen and the methylation was performed using the EPICv1 array.
(iii) An internal set of 15 carcinoma brain metastases: BRCA, LUAD, STAD, UCEC, PRAD, mucinous ovarian cancer (MOC) and CUP. A detailed presentation of these samples can be found in Additional file 1: Table S1.

The study was approved by the ethics commissions of Charité, Universitätsmedizin Berlin (EA1/079/22).

### Tissue microarray construction and immunohistochemistry

For all positive control samples, for which material was available (*n* = 11), we constructed one tissue microarray (TMA) containing three 1.5 mm cores for each tumor. For the other samples (*n* = 5) that were too thin to be included into the TMA we performed whole slide staining. Next, the FFPE TMA and tumor blocks were cut into 2.5 μm sections. One section was used for hematoxylin and eosin (H&E) staining and ten others for p53, SMAD4, GATA6, Ki-67, CK7, CK20, Annexin 1 (ANXA1), Annexin 10 (ANXA10), CD3 and CD20 immunohistochemistry staining. Additionally, the PAAD met.^Liv.^ samples from the biological validation set were stained for p53, SMAD4, GATA6, CK7, CK20, CD3 and CD20. Other immunohistochemistry (IHC) data for the PAAD met.^Liv.^ from the biological validation samples were previously generated [1].

For the immunohistochemical staining, a BenchMark XT immunostainer (Ventana Medical Systems, Tucson, AZ) was used. For antigen retrieval, sections were incubated in CC1 mild buffer (Ventana Medical Systems, Tucson, AZ) for 30 min at 100 °C, or were incubated in protease 1 for 8 min. The sections were stained with anti-Ki-67 antibody (M7240, Dako, 1:50, CC1 mild buffer), anti-p53 (M7001, Dako, 1:50, CC1 buffer), anti-SMAD4 (Ab40759, Abcam, 1:200, CC1 buffer), anti-GATA6 (Q92908, R&D Systems, 1:100), anti-CK7 (M7018, Dako, 1:1000, protease 1), anti-CK20 (M7019, Dako, 1:1000, protease 1), anti-Annexin A10 (PA5-52151, Invitrogen, 1:2000), anti-Annexin I (610066, BD Biosciences, 1:5000), anti-CD3 (A045201-2, Dako, 1:100), and anti-CD8 (M7103, Dako, 1:100) for 60 min at room temperature, and visualized using the avidin–biotin complex method and DAB. We stained the cell nuclei by additionally incubating for 12 min with hematoxylin and bluing reagent (Ventana Medical Systems, Tucson, AZ). Histological images were acquired with the digital slide scanner PANNORAMIC 1000 (3DHISTECH).

### Histological analysis and immunohistochemistry scoring

The ANXA1/10 immunohistochemistry score was proposed as a potential tool for detecting metastatic PAAD. For this purpose, we used the scoring and classification system proposed by Padden et al. [15]. The intensity [0 (none), 1 (weak), 2 (intermediate), or 3 (strong)] and percentage of positive tumor cells [0, 1 (≤ 5%), 2 (6–10%), 3 (11–50%), or 4 (> 50%)] for each tumor was scored separately and the two scores were multiplied, resulting the immunoreactive score (IRS). The IRS thresholds proposed by Padden et al. and validated by us in a previous paper [1] were used also in this study. Hence, an IRS of 5 or higher for Annexin 1 and an IRS of 0.5 or higher for Annexin 10 was suggestive for PAAD. According to the previous studies, only one of the two markers needed to be equal or higher to the IRS cut-off.

For Ki-67 the percentage of positive tumor cells was estimated in representative hot spots. For p53 complete loss or intense nuclear staining were considered to be specific for a mutated pattern. For SMAD4 complete loss was considered to be specific for a mutated pattern. For CK7 and CK20 any degree of cytoplasmic positivity was scored as positive. GATA6 was scored as previously described [16]. Briefly, semiquantitative scoring from 0 (negative) to 4 (intense nuclear) was performed. The samples with scores from 0 to 2 were considered GATA6 low, and the ones with score 3 and 4 were defined as GATA6 high.

We considered an IHC pattern to be specific for PAAD if a tumor showed an ANXA1/10 score that supported the diagnosis of PAAD, and additionally SMAD4 loss and/or CK7 positivity. We considered an IHC pattern to be inconclusive if a tumor showed an ANXA1/10 score that did not support the diagnosis of PAAD, and the tumor showed both SMAD4 loss and CK7 expression, or if the tumor showed an ANXA1/10 score that supported the diagnosis of PAAD, and SMAD4 was expressed and CK7 was negative. We considered an IHC pattern to be unspecific if a tumor showed an ANXA1/10 score that did not support the diagnosis of PAAD, and the tumor showed only SMAD4 loss or CK7 was expressed or none.

### Organoids

Organoids were established from a primary tumor and matched metastases (peritoneal carcinomatosis and liver metastases) from surgical specimens in accordance with the ethics approval EA1/157/21. The tissue was cut into small pieces using scalpels and digested with 100 µg/ml DNAse (STEMCELL Technologies, Vancouver, Canada), 125µg/ml Collagenase II (Sigma-Aldrich, Merck, Darmstadt, Germany), 1:2000 Rock-Inhibitor (Abmole Bioscience, Houston, TX, USA) and 1:200 Amphotericin B (Sigma-Aldrich, Merck, Darmstadt, Germany). The specimens were then incubated for 2 to 3 h. Cells were filtered through a sterile 100µm filter. Red blood cell lysis (Miltenyi Biotec, Bergisch-Gladbach, Germany) was performed if necessary and cells were plated in Cultrex (R&D Systems, Minneapolis, MN, USA). Culture medium as described by Broutier et al. [17] was added after solidification of domes. Amphotericin B was added for the first 7 days of culture to prevent fungal contamination. The culture medium was exchanged every 3 to 4 days and regularly checked for Mycoplasma contamination using the Mycoplasma detection kit (Applied biological Materials, Richmond, Canada). Organoids were split when they reached a size of 200 µm using TrypLE Express (Thermo Fisher Scientific, Waltham, MA, USA) and plated in a ratio of 1:2.

For histologic embedding, organoids were incubated PFA (4% in PBS) at 4 °C. After detachment they were transferred into prewarmed histogel (Thermo Fisher Scientific, Waltham, MA, USA). The organoids were then FFPE. The blocks were cut into 3 μm sections. The slides were stained with H&E using Tissue-Tek Prisma® Plus Automated Slide Stainer (SAKURA). For the immunohistochemical staining, BenchMark XT immunostainer (Ventana Medical Systems, Tucson, AZ) was used. The sections were stained with anti-Ki-67, anti-CA 19-9 (1116-NS-19-9, Dako, 1:500), anti-p53 antibody, and anti-GATA6 antibody. Finally, representative images were acquired with the digital slide scanner PANNORAMIC 1000 (3DHISTECH).

### DNA extraction

For all samples, tumor areas were marked and the tumor cell content was determined using a light microscope (Olympus, BX46). Based on this information we determined the number of necessary slides for DNA extraction. Depending on the tumor purity and tumor surface we used between 7 and 20, 5 μm thick slides per sample from which the tumor contour was scratched for DNA extraction (Additional file 1: Table S2). Semi-automated DNA extraction was performed according to the manufacturer’s instructions (Maxwell RSC FFPE Plus DNA Purification Kit, Custom, Promega). DNA quantities were measured using Qubit HS DNA assay (Thermo Fisher Scientific).

### DNA methylation

Whenever possible we used 500 ng of DNA for the DNA methylation analysis as input. For samples where there was not sufficient material available, we decreased the DNA input to as low as 182.8 ng of DNA. We used the Illumina Infinium HD FFPE DNA Restore Kit (Illumina, CA, USA) for DNA restoration from FFPE samples. Following this step, the EpiTect Bisulfite Kit (Qiagen) was used for bisulfite conversion. For the organoid models we used 100 ng of DNA extracted from fresh tissue. The EPICv1 array was used according to the manufacturer’s instructions for the DNA methylation analysis of the positive and *in-house* negative control samples. The EPICv2 array was used for the hybridization of the 4 PAAD metastases of the technical validation cohort.

### Methylation array processing

Methylation data preprocessing was performed in R using various packages implemented in ChAMP [18]. Raw signals from all the IDAT files are loaded using the minfi package. In the training set, the EPICv1 and the Illumina Infinium HumanMethylation450 BeadChip (Illumina, CA, USA) samples were merged.

In the sample preprocessing for differential methylation analysis, several CpG sites were excluded: those on EPICv1 array not present in 450k arrays; any CpG sites with a detection *p* value greater than 0.01; low quality sites, defined as having fewer than 3 beads in at least 5% of the samples; all SNP-associated sites; multi-hit sites; and CpGs found on chromosomes X and Y.

While preprocessing samples for the classifier, no filtering was necessary, as only the 2048 CpGs which serve as features are selected at the end of the preprocessing pipeline.

Finally, the beta values were extracted and normalized using FunNorm and BMIQ, which together enhanced the process. Each cohort was pre-processed independently.

### t-distributed stochastic neighbor embedding (t-SNE)

To generate the t-SNE plots, beta values of CpG sites were broken down into eigenvectors, and then handled using the R package Rtsne [19] using 5000 iterations. The count of eigenvectors (*k*) and the perplexity (*p*) were chosen individually for each plot to accommodate the varying number of samples.

The t-SNE in Fig. 2A and B were created using 30 eigenvectors (*k* = 30) and a perplexity of 15 (*p* = 15), while for t-SNE in Additional File 1: Fig. S4, we used *k* = 50 and *p* = 20, due to the larger number of samples plotted. The 2048 classifier features served as input for the eigenvector decomposition.

The t-SNE in Fig. 3A consists of 16 primary PAAD, 20 liver and 12 peritoneum metastases unmatched samples. The top 2000 CpGs with the highest standard deviation among these samples were selected and decomposed in 15 eigenvectors (*k* = 15) and then further reduced to two dimensions using t-SNE with a perplexity of 5 (*p* = 5).

### Tumor purity estimation

We estimated the tumor purity using the InfiniumPurify R package [20]. For estimating the purity of iCCA and liver PAAD metastases samples, we selected “CHOL” as tumor type, and “PAAD” as tumor type for the primary PAAD samples and non-liver PAAD metastases.

#### Updated classification pipeline

The anomaly detection layer developed in the previous study could only differentiate between STAD, COAD tumors from PAAD, iCCA and normal bile tumors [1]. This layer has been replaced with an updated version that can separate PAAD, iCCA and normal bile tissue (positive class) from 10 different mimicker carcinomas (negative class).

The new model was built by training a neural network ensemble on the same 2048 CpGs identified in the previous study [1] and used by the classification layer. These are the top 2048 CpGs with the highest standard deviation among the reference samples in the training set. The models were trained using the anomaly detection training dataset consisting of all the reference samples (*n* = 399), which formed the positive class, and 20% of the samples of each of the 10 mimicker carcinomas, selected at random, forming the negative class (*n* = 787).

The model ensemble was built using the python library keras [21] using fourfold cross-validation, while python library optuna [22] was used to conduct hyperparameter optimization. The ensemble consists of 4 neural networks, each network having 5 layers (search space between 1 and 9 layers) with 2048 neurons per layer. A learning rate of 0.0088, a dropout rate of 0.2 (search space: 0, 0.1, 0.15, 0.2, 0.25, 0.3), L1 regularization of 0.00067 (search interval between 0 and 0.1), and 228 epochs (search space between 20 and 300) were found to be the optimal hyperparameters.

The other segments of the pipelines remained as they were in the previous study [1]. The python library reComBat [23] is used to fit a regularized empirical Bayes model to reduce sample storage material induced batch effects. The neural network model in the classification layer was developed in the previous study using python keras and optuna libraries [21, 22]. The optimal network was found to consist of 8 layers (search space 1 and 10 layers) with a starting width of 256 neurons (search space 64, 128, 256, 512, 1024, 2048, 4096) for the first hidden layer, incrementally decreasing to 16 neurons in the last hidden layer. It was trained with a learning rate of 0.00895 (search space between 0.0001 and 0.01). Finally, a dropout rate of 0 (search space 0, 0.1, 0.15, 0.2, 0.25, 0.3), L1 regularization of 0.00441 (search space between 0 and 0.1), and 191 epochs (search space between 20 and 300) were found to perform optimally. To further increase the accuracy and confidence in the model’s output, a threshold of 0.8 for the PAAD and iCCA classes and 0.5 for the normal bile were selected. Predictions which did not reach the threshold were put into the “No Match” class, together with the samples rejected by the anomaly detection layer described above.

The updated classification pipeline is therefore composed of three parts: (i) the anomaly detection layer that singles out PAAD, iCCA, and normal bile tissue from other carcinomas; (ii) the classification layer capable of differentiating between PAAD, iCCA, and normal bile samples; and (iii) a threshold-based filtering layer that weeds out samples with low confidence predictions. The result can therefore belong to one of four classes: PAAD, iCCA, normal bile tissue, or “No Match”. The “No Match” class contains all the samples rejected by the anomaly detection layer and the samples that passed the anomaly detection layer but did not reach the level required by the threshold-based filtering layer.

#### Copy number analysis

We calculated the copy number profiles from DNA methylation array data using the conumee package, version: 1.3.0. [24]. A set of 63 control samples derived from histologically confirmed normal pancreas tissue were used as baseline reference. The evaluation of copy number alterations was carried out manually with consideration of the tumor cell content for the evaluation of chromosomal gains or losses. In general, changes were considered relevant if the intensity ratio of a segment deviated from the baseline by at least more than 0.15 [25]. In addition, we created summary copy number profiles for three different groups: primary PAAD (*n* = 16), PAAD met.^PC^ (*n* = 11), and PAAD met.^Liv.^ (*n* = 21). This analysis was done using an adaptation of the conumee script (provided by Dr. Damian Stichel, Neuropathology Heidelberg). For the comparison of specific gene deletions and amplifications between the three groups we performed Fisher's exact test with Bonferroni correction for multiple testing.

#### Differentially methylated CpG probes and pathway analysis

The differentially methylated analysis (DMA) followed by pathway analysis was conducted on 48 samples from 3 groups, 16 primary PAAD tumors, 21 PAAD met.^Liv.^ and 11 PAAD met.^PC^. The R package limma as implemented in ChAMP was used. Limma deploys a linear model alongside an empirical Bayes approach to gauge the mean methylation disparity between groups, following which it computes adjusted *p* values to accommodate multiple testing. An adjusted *p* value below 0.01 and an absolute logFC value exceeding 0.2 were chosen as thresholds to select the differentially methylated CpGs between groups.

We used the Illumina Infinium HumanMethylationEPIC manifest to annotate promoter and enhancer CpGs. Genes associated with differentially methylated promoter- and enhancer-associated CpGs between groups (primary PAAD, PAAD met.^Liv.^, and PAAD met.^PC^) were analyzed for enriched pathways using Enrichr [26]. The Reactome (2022) Pathway Database was selected for the enrichment analysis. Volcano plots were created using VolcaNoseR [27]. From the top 10 enriched pathways we labeled the ones linked to epithelial and mesenchymal phenotypes. In addition, we used a second method for pathway analysis, methylGSA [28]. We used the same genes associated with differentially methylated promoter- and enhancer-associated CpGs between the three groups for this analysis. The KEGG pathway database was selected for the tested gene sets.

#### Classifier website

The website is a Vue.js application built with Nuxt.js running on a Node.js platform. It utilizes client-side rendering and leverages Google Firebase for secure user authentication. The application backend responsible for processing the raw data and running the prediction was developed in python using the FastAPI framework. The application is hosted on Google Cloud.

#### Statistical analysis

We performed statistical analyses and graphics using the GraphPad Prism 9 software. First, we determined whether the data followed a normal distribution, using the Shapiro–Wilk normality test. For the comparison between two groups, p‐values were determined with an unpaired t test if the data were normally distributed, while the nonparametric Mann–Whitney–Wilcoxon test was applied on data with a non‐normal distribution. For the comparison between multiple groups p‐values were determined using ordinary ANOVA test for normally distributed data, and the Kruskal–Wallis test for data with a non‐normal distribution. Correlations were performed using the Pearson correlation test. All tests were two‐sided, and a *p* value < 0.05 was considered statistically significant.

## Results

### Updated *PAAD-iCCA-classifier* performance

**Fig. 1:**
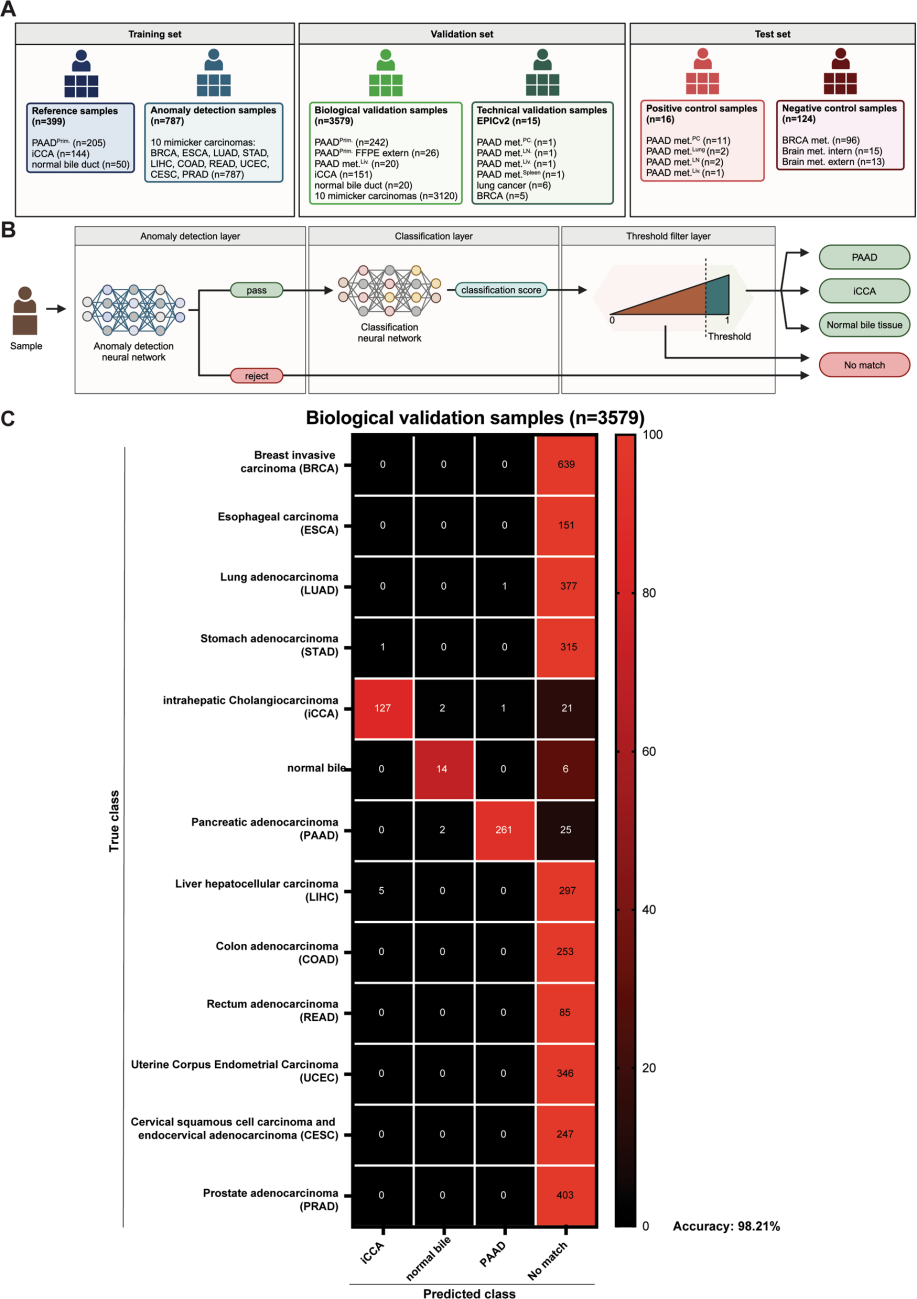
Upgrading the classifier. **A** Overview of the patient datasets used to develop, validate and test the classifier. **B** Overview of the classification pipeline. **C** Confusion matrix with the classifier results after applying the anomaly detection filter and the specific thresholds for the biological validation samples (*n* = 3579)

The original version of the *PAAD-iCCA-Classifier* was designed with an anomaly detection layer trained only on STAD and COAD [1]. Therefore, we first enlarged our anomaly detection layer with other carcinomas that could come into question in a PAAD metastatic setting by adding 8 additional carcinomas from the TCGA methylation datasets: BRCA, ESCA (both adenocarcinomas and squamous carcinomas), LUAD, LIHC, READ, UCEC, CESC, and PRAD, reaching a total of 787 samples (Fig. 1A). Next, we validated the classifier on a biological and on a technical validation sample group, and lastly tested it on extrahepatic PAAD metastases and various non-PAAD metastases as negative controls (Fig. 1A). Briefly, each sample entering the classification process needed to pass the anomaly detection layer in order to enter the classification layer, and then needed to pass specific thresholds to be classified as PAAD, iCCA or normal bile duct tissue. If excluded at any point, the sample would be labeled as “No Match” (Fig. 1B).

We first analyzed the anomaly detection layer on the biological validation cohort and observed that out of 3120 mimicker carcinomas samples, only 12 (0.38%) obtained a positive result and passed the anomaly detection layer, most of them being LIHC (*n* = 5) (Additional file 1: Fig. S1A and Additional file 2: Table S3). As expected, nearly all the positive class samples passed this layer: 141/151 (93.38%) iCCAs, 20/20 (100%) normal bile ducts, and 276/288 (95.83%) PAADs. Overall the anomaly detection layer achieved an accuracy of 99.05% (Additional file 1: Fig. S1A).

We then introduced the biological validation samples to the classification layer capable of distinguishing between iCCA, normal bile duct, PAAD, and “No Match” and achieved an accuracy of 98.21%. We observed that one sample from the mimicker carcinomas group was classified as PAAD, more specifically a LUAD sample, and one iCCA sample was also classified as PAAD. On the other hand, seven LIHC samples and one STAD sample were misclassified as iCCA (Fig. 1C and Additional file 2: Table S3). Altogether, this data supports the hypothesis that the *PAAD-iCCA-Classifier* could be used to diagnose PAAD metastases from extrahepatic sites. In addition, we also verified the effect of tissue material (FFPE vs. fresh frozen) on the classifier performance, and as previously observed [1], we achieved a higher true class score on fresh frozen tissue compared to FFPE (Additional file 1: Fig. S1B).

Recently, a new generation of DNA methylation array chips was released (Infinium human MethylationEPICv2 BeadChip), and we considered that for the future implementation of the classifier, it is essential to also validate it on samples analyzed using these new chips (technical validation). For this purpose, we analyzed 6 lung cancers and 5 BRCA from GSE222919, and 4 *in-house* hybridized PAAD metastases. All BRCA and lung cancer samples failed to pass the anomaly detection filter and were classified as “No Match” by the classifier, while the PAAD met. samples were correctly labeled as PAAD (Additional file 1: Fig. S1C, D and Additional file 2: Table S3).

### Off label use of the classifier

To confirm the PAAD origin of the positive control samples we performed a broad histological and IHC analysis of the 16 patients with highly suspected or confirmed PAAD metastases (Additional file 1: Table S4). Of these patients, 11 had peritoneal carcinomatosis (Additional file 1: Fig. S2A), two had lung metastases (Additional file 1: Fig. S2B), two had indirect abdominal lymph node metastases (truncus coeliacus) (Additional file 1: Fig. S2C), and one had liver metastases (Additional file 1: Fig. S2D). Twelve of the patients had synchronous metastases with four undergoing simultaneous pancreas resections with confirmed PAAD histology, while the other eight patients had an imagistic suspect infiltrative mass in the pancreas. Three other patients had metachronous metastases with a previously resected primary PAAD. For the one remaining patient, we were not able to determine if the metastasis was synchronous or metachronous but an imagistic suspect infiltrative mass in the pancreas further supported the diagnosis of PAAD metastasis. Histologically, nine of the tumors were still forming glands, four of which had a conventional morphology, while the other five had a tubular-papillary morphology. Seven other tumors did not form glands and showed a composite morphology. Twelve of the tumors were moderately-differentiated and the other four were poorly-differentiated (Additional file 1: Table S5). Immunohistochemically, eight tumors showed a p53 mutated pattern, and only three showed a SMAD4 mutated pattern. We next performed a semiquantitative assessment of GATA6 [16] and observed that 14 metastases had a high GATA6 score (classical subtype) and the other two a low score (basal like subtype). The average proliferation rate of the metastatic tumors was 31%. Regarding the cytokeratin expression pattern, ten tumors were CK7^+^/CK20^+^, five were CK7^+^ /CK20^−^, one was CK7^−^/CK20^+^, and none was CK7^−^/CK20^−^. Using the ANXA1/10 scores [15], we observed that 13 metastases were above the thresholds, hence supporting the diagnosis of PAAD, while 3 were below the thresholds. This data together further supports the diagnosis of PAAD for our samples but also outlines the limits of IHC for the diagnosis of PAAD. Collectively, of the 16 patients, 12 show IHC that supports the diagnosis of PAAD, two have an inconclusive IHC pattern and two an unspecific IHC pattern (Additional file 1: Fig. S2E).

**Fig. 2:**
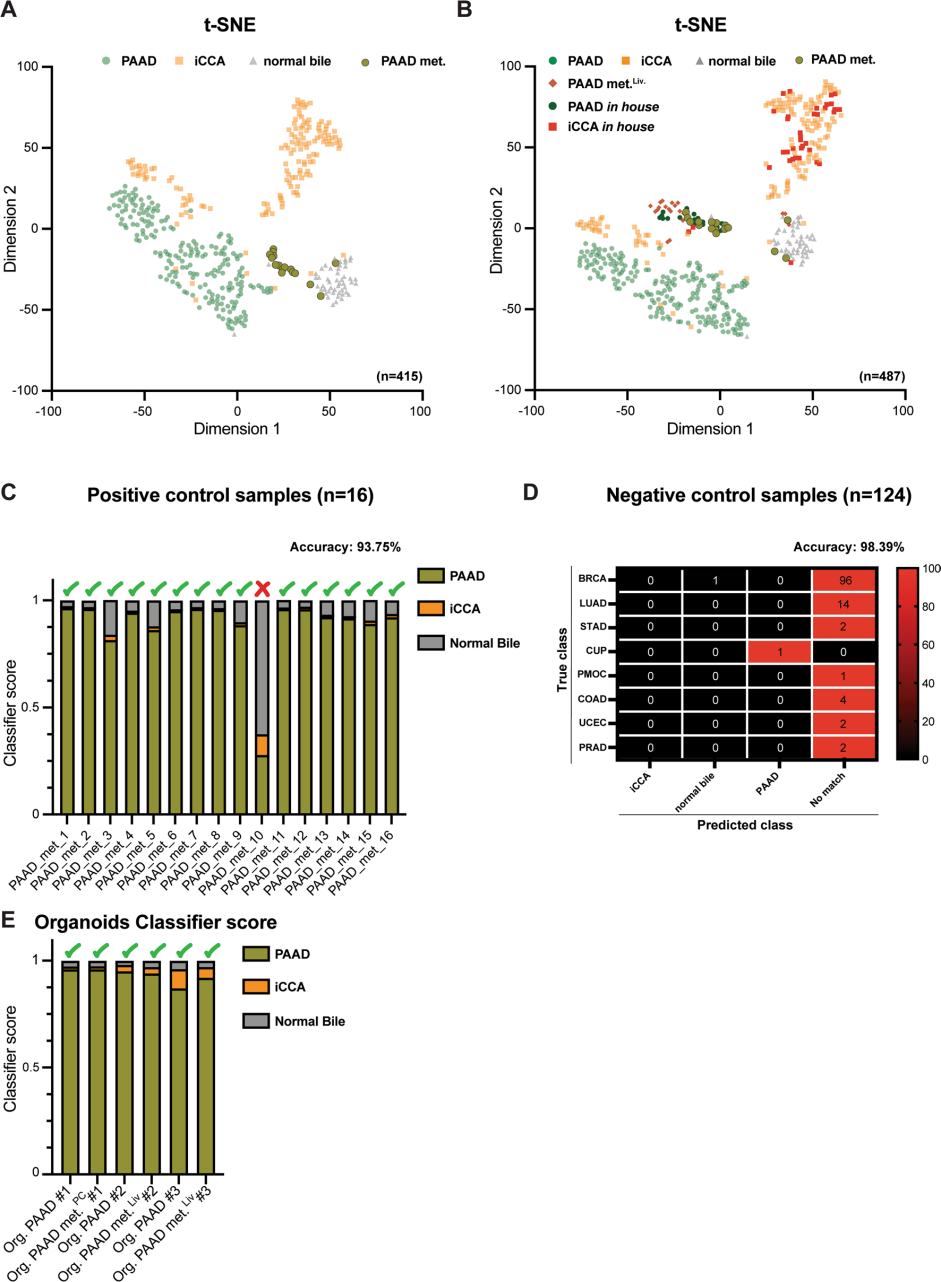
Off-label use of the classifier. **A** The two-dimensional representation using the t-SNE method based on the DNA methylation profiles of the reference cohort used to develop the classifier, to which the PAAD metastasis positive control samples were added (*n* = 415). **B** The two-dimensional representation using the t-SNE method based on the DNA methylation profiles of the reference cohort to which we added all *in-house* PAAD samples and *in-house* iCCA samples from the biological validation and the positive control samples (*n* = 487). **C** Classifier results of the positive control samples. **D** Confusion matrix with the classifier results after applying the anomaly detection filter and the specific thresholds for the negative control samples (*n* = 124, from three independent cohorts: BRCA metastases from 18 anatomical sites, *n* = 96; brain metastases extern, *n* = 13; brain metastases intern, *n* = 15). **E** Classifier results of the organoid models

Next, we wanted to test if the *PAAD-iCCA-Classifier* can also be used to classify PAAD samples with extrahepatic localization. We visualized the 16 positive control samples together with the reference samples by performing a t-SNE analysis (n = 415 individual biological samples). As expected, 13/16 samples of the positive control were located close to the PAAD group, while the other 3 grouped together with the normal bile duct samples (Fig. 2A). The localization close to PAAD and not with the PAAD samples of the reference cohort could mainly be attributed to the fact that the positive control samples were FFPE and hybridized with EPICv1, while the PAAD reference samples were fresh frozen and analyzed with the 450k array, implying some batch effects (Additional file 1: Fig. S3A-C). In order to test this hypothesis, we performed a second t-SNE with additional PAAD FFPE samples from our previous publication. Indeed, we observed that both primary PAAD and PAAD liver metastases were localized closely to our PAAD positive control samples, suggesting local relationships (Fig. 2B). Also, in this second t-SNE analysis, the same three samples fell into the normal bile duct tissue group. These three samples were metastases from different organs: liver, lung, and lymph node (Additional file 1: Fig. S3D). We observed that one of the three samples showed an unspecific IHC profile for PAAD, while the other two showed a specific profile (Additional file 1: Fig. S3E). Regarding tumor purity, we observed that the three samples that are grouped with normal bile duct tissue have one of the lowest tumor purities of the positive control cohort (Additional file 1: Fig. S3F).

We also visualized our positive control samples against the training set. In the t-SNE plot, we observed that the positive control ones were positioned among the PAAD samples, indicating local relationships. Additionally, some were positioned near the normal bile duct tissue samples, suggesting local similarity in this context (Additional file 1: Fig. S4A). Generally, this data suggested that there may be a risk to classify some PAAD metastases as normal bile duct tissue but not as other carcinomas.

We went on to test the neural network classifier on the 16 positive control samples and observed that 15/16 were correctly classified as PAAD (accuracy 93.75%). The only misclassified sample (sample #10) was as expected classified as normal bile duct tissue (Fig. 2C and Additional file 2: Table S3). Sample #10 is one of the two PAAD lung metastases, being located in the t-SNE analysis together with normal bile tissue samples. Additionally, this sample showed one of the lowest tumor purities.

The negative control permitted us to test the impact of different tissue types on our classifier. Of all samples, 11/124 (91.13%) passed the anomaly detection layer (Additional file 1: Fig. S4B and Additional file 2: Table S3). This group consisted of 2 BRCA lymph node metastases, suggesting a negative impact of the immune infiltrate on the anomaly detection filter, 6 LUAD, 1 STAD and 1 CUP sample (probable of upper GI tract origin based on IHC), suggesting that the anomaly detection filter is impacted by samples originating from the embryonic foregut, and 1 MOC sample, for which the anomaly detection was never trained.

Looking at the whole classification pipeline results, only two passed the threshold, one being labeled as normal bile duct and the other one as PAAD (98.39% accuracy, Fig. 2D and Additional file 2: Table S3). Interestingly, the sample misclassified as normal bile was a BRCA lymph node metastasis, further suggesting that the immune infiltrate impacts the results of the classifier, while the sample classified as PAAD was the CUP sample from the internal brain metastases cohort, a sample for which the IHC suggests upper GI tract origin, and for which PAAD origin cannot be excluded.

In our previous work using indirect deconvolution methods [1] and now by analyzing our negative control samples, we observed that the results of the classifier are mainly influenced by the immune infiltrate. Therefore, for the positive control (*n* = 16) and for the metastatic samples from the biological validation cohort (*n* = 20 PAAD met.^Liv.^, Additional file 1: Table S6), we correlated the predicted score of the correct class with CD3^+^ and CD20^+^ immune infiltrate, tumor purity, and proliferation rate (Ki-67%). We observed a negative correlation for the more abundant CD3^+^ immune infiltrate (Pearson *r* = −0.1579, *p* = 0.39), but not for CD20^+^ (Pearson *r* = 0.099, *p* = 0.59) with the predicted score for the correct class (Additional file 1: Fig. S5A-B). On the other hand, a positive correlation was observed between the proliferation rate (Pearson *r* = 0.2377, *p* = 0.16), tumor purity (Pearson r = 0.2755, *p* = 0.1) and the predicted score of the correct class (Additional file 1: Fig. S5C-D). We then examined whether there was an association between the predicted score of the correct class and metastatic site (*p* = 0.97), morphology (*p* = 0.86), grade (*p* = 0.92), p53 status (*p* = 0.14), SMAD4 status (*p* = 0.29), GATA6 score (*p* = 0.95), CK7/20 profile (*p* = 0.68), ANXA score (*p* = 0.93), and IHC score (*p* = 0.99). No significant differences were observed for any of the comparisons (Additional file 1: Fig. S5E-M).

Next, we wanted to check if the classifier could correctly label organoids from primary and metastatic tumors that grew in a completely unnatural environment. We performed DNA methylation on three pairs of organoids from primary and metastatic PAAD (one PAAD met.^PC^ and two PAAD met.^Liv.^). We confirmed that the organoids were from tumor tissue by analyzing the IHC and copy number alteration (CNA) profile (Additional file 1: Fig. S5N-O). Finally, both the primary and metastatic PAAD organoids passed the anomaly detection and were classified with very high accuracy as PAAD by the classifier (scores ranging between 0.87 and 0.96 for PAAD) (Fig. 2E).

### DNA methylation-associated organotropism of pancreatic adenocarcinoma metastases

We wanted to use this unique dataset to gain a better understanding of the genome-wide DNA methylation differences that exist between PAAD samples that have metastasized to the liver (*n* = 21 PAAD met.^Liv.^), PAAD samples that have spread into the peritoneal cavity (*n* = 11 PAAD met.^PC^), and primary PAAD samples that showed no evidence of metastatic disease (*n* = 16, primary PAAD).

First, we performed CNA profiles using DNA methylation data for each of the three groups. We observed that most of the PAAD met.^Liv.^ samples showed a similar profile, characterized by ample chromosomal deletions, including chr. 6, chr. 8p, chr. 9 and chr. 18q deletions (Additional file 1: Fig. S6A). On the other hand, primary PAAD and PAAD met.^PC^ samples did not show a characteristic CNA profile (Additional file 1: Fig. S6B and C). Regarding gene deletions we observed additional differences between the groups. For example, *CDKN2A* deletion was significantly more common in PAAD met.^PC^, with 100% of the samples showing deletions compared to 63% in primary PAAD and 52% in PAAD met.^Liv.^ (Fisher’s exact test with Bonferroni correction, adjusted *p* = 0.027). *SMAD4* deletion was a chromosomal alteration specific only to PAAD met.^Liv.^ being deleted in 29% of this group and in none of the cases from the other groups (Fisher’s exact test with Bonferroni correction, adjusted *p* = 0.027).

**Fig. 3:**
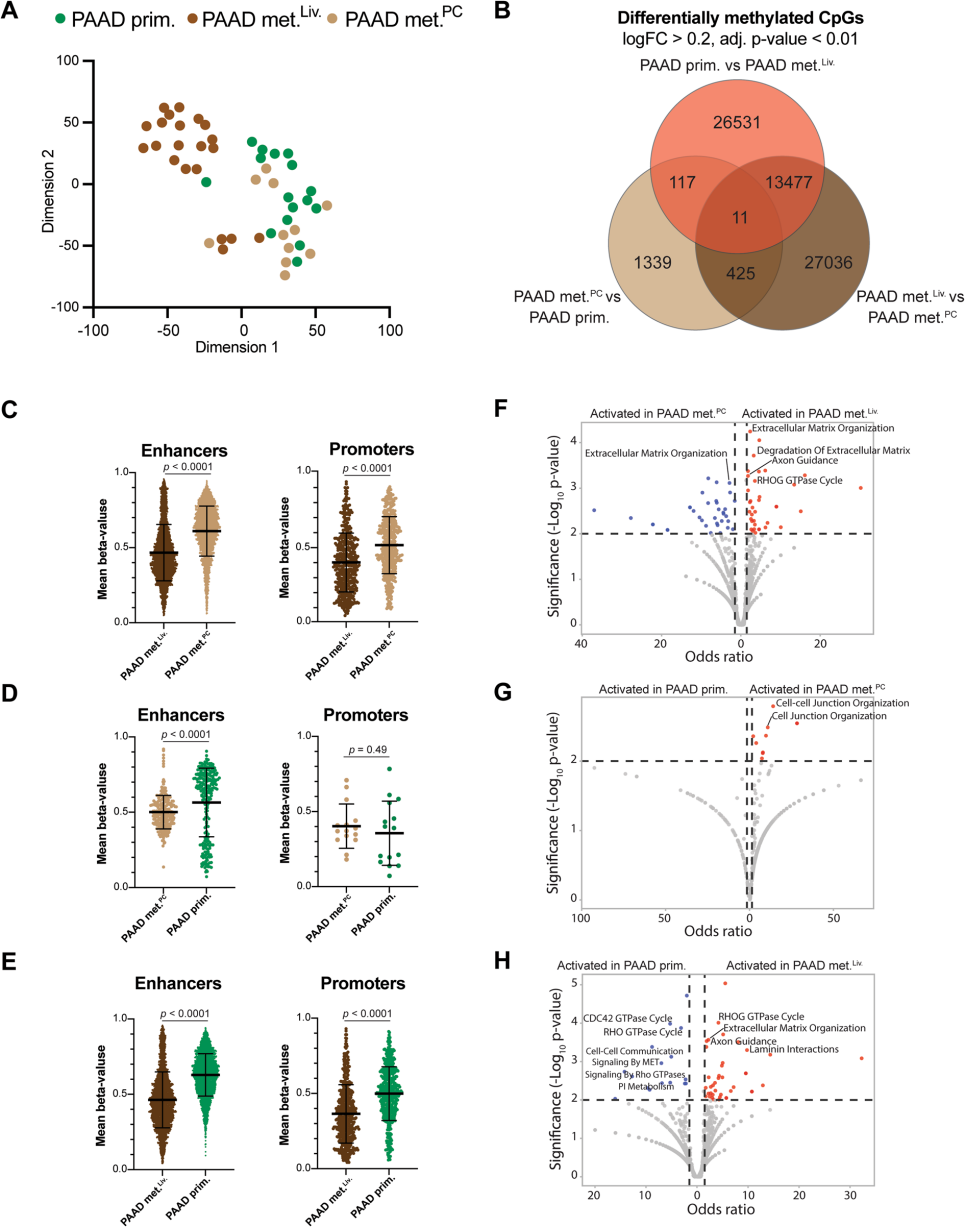
DNA methylation-associated organotropism of pancreatic adenocarcinoma metastases. **A** The two-dimensional representation using the t-SNE method, based on the DNA methylation profiles of *in-house* primary PAAD (*n* = 16), PAAD met.^PC^ (*n* = 11), and PAAD met.^Liv.^ (*n* = 21). **B** Venn diagram comparing differentially methylated CpGs (log FC > 0.2, and adj. *p* value < 0.01) between primary PAAD and PAAD met.^Liv.^ and PAAD met.^PC^ Comparison of enhancer- (left) and promoter-associated (right) DNA methylation levels (beta values) in **C** PAAD met.^Liv.^ versus PAAD met.^PC^, **D** PAAD met.^PC^ versus primary PAAD, and **E** PAAD met.^Liv.^ versus primary PAAD. Volcano plots showing significantly activated pathways of genes linked to enhancer-associated CpGs (hypomethylated) in **F** PAAD met.^Liv.^ versus PAAD met.^PC^, **G** PAAD met.^PC^ versus primary PAAD, and **H** PAAD met.^Liv.^ versus primary PAAD

Secondly, we visualized the samples using t-SNE, and observed that PAAD met.^Liv.^ grouped separately, while most of the PAAD met.^PC^ grouped with primary PAAD (Fig. 3A).

Thirdly, we performed DMA and observed more differentially methylated CpGs between PAAD met.^Liv.^ and PAAD met.^PC^ or primary PAAD than between PAAD met.^PC^ and primary PAAD (Fig. 3B).

Next, a more detailed analysis of enhancer- and promoter-associated differentially methylated CpGs revealed that these are significantly more hypomethylated in PAAD met.^Liv.^ compared to PAAD met.^PC^ or primary PAAD, whereas only enhancer-associated CpGs are hypomethylated in PAAD met.^PC^ compared to primary PAAD (Fig. 3C-E). These CpGs were mapped to more enhancers than promoters, revealing 891 versus 248 genes associated with hypermethylated enhancer-associated CpGs in primary versus liver metastases. For PAAD met.^Liv.^ versus PAAD met.^PC^ we obtained 356 genes with enhancer-associated hypermethylated CpGs in liver metastasis samples versus 948 genes with enhancer-associated hypermethylated CpGs in PAAD met.^PC^. While for primary PAAD versus PAAD met.^PC^ the differences were less striking, as we found 76 genes with enhancer-associated hypermethylated CpGs in primary versus 28 in PAAD met.^PC^ (Additional file 3: Table S7). We then performed pathway enrichment analysis using these gene sets. Comparing PAAD met.^Liv.^ to PAAD met.^PC^, we observed that hypomethylated enhancer genes (i.e. transcriptionally active genes) in PAAD met.^Liv.^ were frequently associated with pathways involved in epithelial-mesenchymal transition (EMT), such as: Extracellular Matrix Organization R-HSA-1474244, Degradation Of Extracellular Matrix R-HSA-1474228, Axon Guidance R-HSA-422475, and RHOG GTPase Cycle R-HSA-9013408 (Fig. 3F, Additional file 4: Table S8). When comparing PAAD met.^PC^ with primary PAAD only small differences were noticed, revealing the activation in PAAD met.^PC^ of EMT pathways associated with Cell-cell Junction Organization R-HSA-421270, and Cell Junction Organization R-HSA-446728 (Fig. 3G). Finally, several different EMT pathways were differentially activated in PAAD met.^Liv.^ versus primary PAAD, with RHOG GTPase Cycle R-HSA-9013408, Extracellular Matrix Organization R-HSA-1474244, Axon Guidance R-HSA-422475, and Laminin Interactions R-HSA-3000157 activated in PAAD met.^Liv.^ (Fig. 3H). Lastly, we performed an additional methylation specific pathway analysis of promoter and enhancer associated differentially methylated CpG probes using methylGSA [28]. When performing the analysis for primary PAAD versus PAAD met.^PC^ there was no pathway where we achieved a ratio between detected genes and total pathway genes of 10% (highest ratio = 3.79%). For the comparison PAAD met.^Liv.^ versus PAAD met.^PC^ we detected 8 pathways reaching a ratio of 10% or higher and between the top hits we detected Axon guidance and Focal adhesion (Additional file 1: Fig. S7A). When performing the comparison for PAAD met.^Liv.^ versus primary PAAD we detected 6 pathways reaching a ratio of 10% with the top two hits being the previously detected pathways Axon guidance and Focal adhesion (Additional file 1: Fig. S7B). This additional analysis suggests that PAAD met.^Liv.^ exhibits a distinct epigenetic profile compared to primary PAAD and PAAD met.^PC^, with potential involvement in the EMT process.

Overall, these data indicate that PAAD met.^Liv.^ may present a more mesenchymal profile from a DNA methylation perspective, which could reflect characteristics of metastasis, while primary PAAD and PAAD met.^PC^ show greater epigenetic similarity.

## Discussion

Herein, we show that the *PAAD-iCCA-Classifier* can be used for the diagnosis of PAAD metastases with various locations. The main purpose of this improved version of the classifier was to increase the safety of the tool, as the probability of encountering other carcinomas as input increases. This can be addressed in two ways: by setting thresholds for final scores [29–31] or by creating an anomaly detection filter [12]. To increase the security of our classifier, we combined both methods.

Regarding the use of this classifier which is now available online (at https://classifier.tgc-research.de/), we would like to point out several particularities. First, since most PAAD metastases are small, have low tumor purity and a very desmoplastic background stroma, we observed that better results were obtained by scratching serial slides compared to punching out regions of interest.

**Fig. 4:**
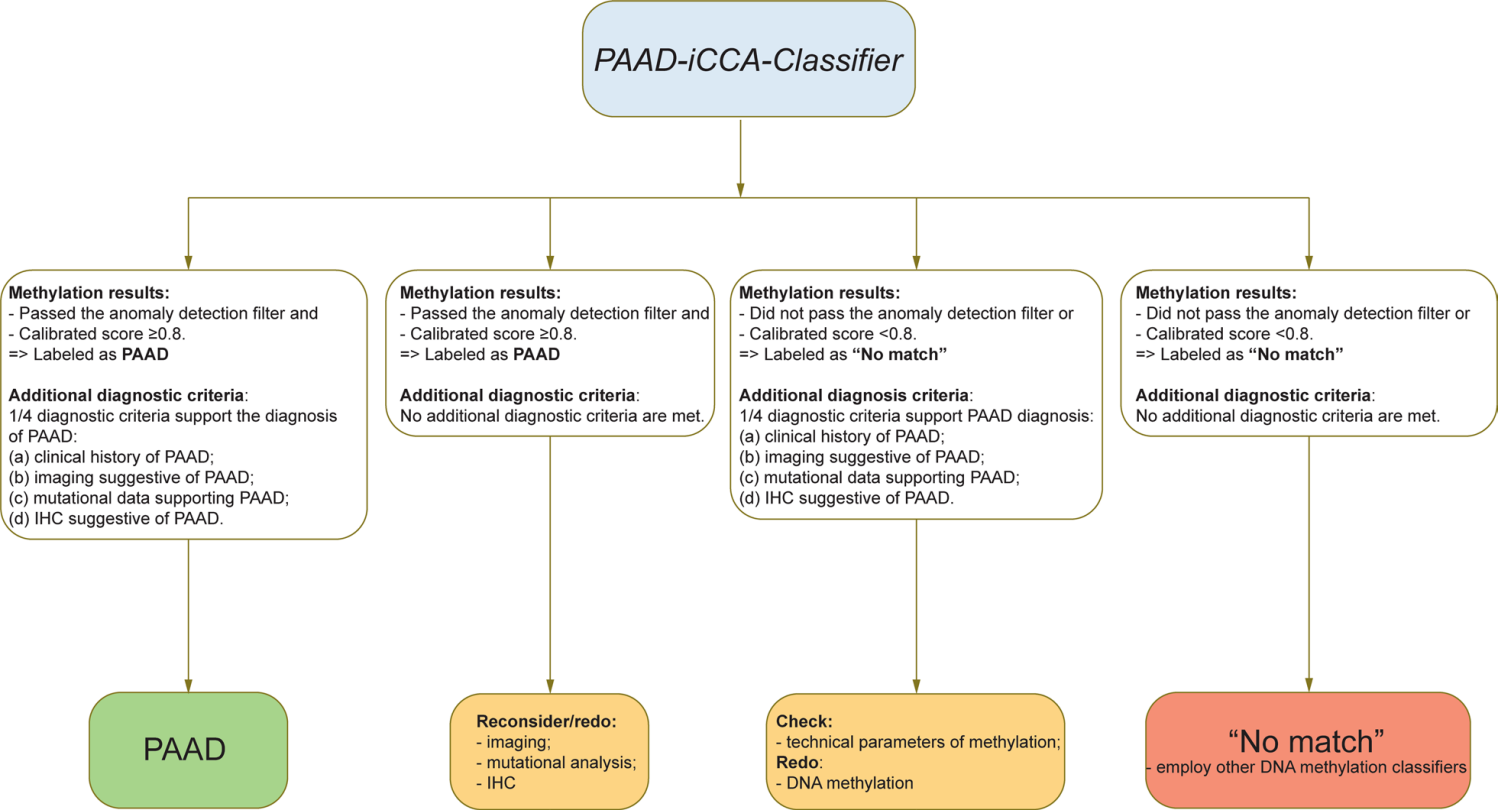
DNA methylation workflow for the *PAAD-iCCA-Classifier.* Proposed workflow for the *PAAD-iCCA-Classifier* for PAAD samples in a CUP-like scenario

Second, because of the vast possibilities of input entities and because there is no highly specific IHC for PAAD, the workflow for translating the results of *PAAD-iCCA-Classifier* into diagnosis will have some particularities (Fig. 4) compared to other classifiers [32]. We consider that in order for a tumor to be diagnosed as PAAD the sample should pass the anomaly detection filter, receive a prediction with a probability ≥ 0.8 from the neural network classifier, and additionally, the diagnosis should be supported by at least one of the following: (a) clinical history of PAAD, (b) matching imaging data, (c) mutational data suggesting the diagnosis, or (d) IHC suggesting the diagnosis of PAAD: SMAD4 loss and or ANXA10 overexpression. For cases where the methylation score indicates PAAD, but no second criterion is reached, we recommend redoing imaging, mutational analysis and/or immunohistochemistry from a second FFPE block if possible. For the scenario where the history/imaging/mutational data or IHC suggests PAAD, but the classifier returns “No Match”, one should check the sample for immune infiltrate, low tumor purity, and proliferation and grading, and based on these consider redoing the DNA methylation analysis, if possible, from a second FFPE region/block. Indeed, in our initial publication we observed that a higher immune cell presence within the tumor was associated with lower confidence in the classifier’s prediction [1]. This finding came from performing cell deconvolution on bulk methylation data to estimate the proportions of various cell types. Since this method did not distinguish between different immune cell types, in the current study we further analyzed the correlation between the number of positive CD3 and CD20 cells with the predicted class score. As CD3 cells were present in much larger amounts compared to CD20, a negative correlation was observed between classifier score and CD3, but not for CD20. Similarly, when looking at the relation between the tumor proliferation rate (Ki-67) and the prediction score, we observed that a higher proliferation rate was associated with an increase in the classifier’s prediction confidence. Finally, we also checked if the classifier can correctly label organoids from primary and metastatic PAAD tumors. In this way we analyzed if an unnatural environment that could induce epigenetic changes, induced by factors such as cell culture media, could impact the results of the classifier. We observed that all organoids were correctly classified despite this unnatural environment.

For the situation in which the classifier output is “No Match” and no additional criteria suggests PAAD, one should consider other differential diagnoses and potentially use the DNA methylation profile to plot the sample in other DNA methylation classifiers [12, 29–31].

Finally, we acknowledge that the *PAAD-iCCA-Classifier* most probably cannot exclude ampullary adenocarcinomas or distal cholangiocellular carcinomas and would probably label these tumors as PAAD or iCCA. However, we do not perceive this as an important limitation mainly because these tumors are diagnosed and treated surgically very similar to PAAD.

This is not the first classifier designed to detect PAAD. Draškovič and Hauptman built a model capable of recognizing BRCA, LIHC, LUAD, PAAD, STAD, CCA, and COAD and READ, as well as liver metastases of PAAD and BRCA [33]. Their focus was on differentiating primary tumors, which they achieved with high precision (85.3–96.4% accuracy). When tested on 13 PAAD liver metastases, their classifier demonstrated an accuracy range of 86.8–94.5%. This shows that high diagnostic accuracies can be achieved through methylation profiling. In another paper, Bai et al. determined a biomarker profile of methylation sites specific to primary liver cancer and its subtypes, LIHC and iCCA [34]. They built a random forest algorithm which achieved high accuracy in detecting primary liver cancer (97.3% sensitivity, 81% specificity) and in separating LIHC from iCCA (96.8% LIHC and 85.4% iCCA accuracy). These results are comparable to the high accuracy of our anomaly filter in distinguishing iCCA from LIHC. Another classifier relying on a random forest algorithm is the EPICUP. Built by Moran et al. it was designed to identify the primary sites of CUP [35]. The classifier was trained on 38 tumor types and predicted the primary site of 188 out of 216 (87%) patient-test-set cases, 14 (7%) of which were PAAD metastases. As it was designed for a broad spectrum of cancers, it only included 100 primary PAAD in its training set and 57 PAAD samples in the validation cohort, of which 9 were metastases.

Our classifier, developed on 205 primary PAAD samples and validated on 268, was tested on a larger cohort of PAAD metastases (*n* = 40) and includes a broader range of metastatic sites (*n* = 5) than previous studies. Additionally, our approach incorporates an anomaly detection filter, a threshold filter and a mandatory set of criteria that must be met before confirmation of the diagnosis as these are necessary steps in order to build a classifier that can be used as an aid in clinical practice. Finally, we developed a web tool to facilitate easy access to our classifier: https://classifier.tgc-research.de/. For future research and using the same strict criteria, we are planning to expand the classifier onto other types of adenocarcinomas.

In addition, we used this unique genome wide DNA methylation data to perform an exploratory analysis of the epigenetic program of primary un-metastasized PAAD, PAAD met.^PC^ and PAAD met.^Liv.^. Our exploratory analysis suggests that PAAD met.^Liv.^ differs from primary PAAD and PAAD met.^PC^, displaying a specific CNA profile and a general hypomethylation of enhancers and promoters. In contrast, primary PAAD and PAAD met.^PC^ appear somewhat similar from an epigenetic standpoint. Our results align with recent in vivo data showing that liver metastases of PAAD is accompanied by an important enhancer reprogramming. Similar to our data the enhancer reprogramming leads to an activation of mesenchymal programs [36]. The already existing data on this topic used enhancer markers (H3K27ac) for revealing the mechanism of enhancer reprogramming by analyzing patient derived organoids [36], hence we bring a new perspective on this topic, showing that enhancer-associated CpGs are hypomethylated in liver metastases. The observation that hematogenous metastases are different from peritoneal metastases (carcinomatosis) is not new and is confirmed both experimentally (PAAD met.^PC^ cells have no increased capacity to spread hematogenous) [37, 38] and at the molecular level [39]. What was surprising to us was that PAAD met.^PC^ was more similar to primary resectable PAAD than PAAD met.^Liv.^. One potential trivial explanation for this observation is that an important step of a peritoneal spread is that the tumor reaches the peritoneal surface being followed by spontaneous exfoliation of tumor cells [40]. Therefore, in the very small retroperitoneal region of the pancreas, the most important difference between a resectable PAAD and a PAAD with peritoneal spread can be as little as a few millimeters in size. Future studies focusing on other omics and the tumor microenvironment are needed to further confirm this observation. For example, single-cell RNA sequencing analysis of 11 PAAD patients recently demonstrated that a specific tumor microenvironment (TME) is responsible for liver metastasis [41].

Our study has several limitations that must be acknowledged. First, our positive control cohort includes only 16 metastatic PAAD samples. It should be noted that PAAD metastases are almost never resected and very little material is available for research. In addition, for the exploratory mechanistic studies we added an additional 20 PAAD met.^Liv.^. Second, for both the classifier analysis and the exploratory part, it would have been helpful to have matched samples, primary and metastatic tumor from the same patient. Third, this classifier is developed retrospectively and needs to be validated in a prospective clinical scenario.

## Conclusion

We show that the *PAAD-iCCA-Classifier* can be used to diagnose PAAD samples with high accuracy regardless of the site of metastasis. Furthermore, exploratory mechanistic data reveal that from an epigenetic perspective, PAAD met.^Liv.^ have a profile characterized by specific CNA and hypomethylation of enhancers involved in the EMT process. This epigenetic program differs from that of primary PAAD and PAAD met.^PC^, which show a more similar DNA methylation profile.

## Supplementary Information

Supplementary Material 1: Figure S1. Upgrading the classifier. **A** Confusion matrix with the results of the anomaly detection layer for the biological validation samples (*n* = 3579). **B** Comparison of the probability score of the correct class between fresh frozen and FFPE tissue in the validation cohort. **C** Confusion matrix with the results of the anomaly detection layer for the technical validation samples (*n* = 15). **D** Confusion matrix with the classifier results of the technical validation samples - EPICv2 (*n* = 15). Figure S2. Characterization of the positive control samples. **A** Examples of H&E and IHC staining of peritoneal carcinomatosis from PAAD, **B** PAAD lung metastasis, **C** PAAD lymph node metastasis, and **D** PAAD liver metastasis. **E** Overview of the patient characteristics. Figure S3. t-SNE analysis of the reference and positive control samples. The two-dimensional representation of the reference cohort and positive control samples (*n* = 415) using the t-SNE method based on DNA methylation profiles. The color code of the samples represents: **A** the origin of the study set, **B** material type, **C** array type, **D** metastases origin, **E** IHC profile suggestive for, and **F** tumor purity. Figure S4. Off label use of the classifier. **A** The two-dimensional plot representation using the t-SNE method, based on the DNA methylation profiles of the positive control group (*n* = 16) together with reference samples (*n* = 399) and anomaly detection samples (10 different carcinomas, *n* = 787). BRCA—breast invasive carcinoma, ESCA—esophageal carcinoma, LUAD—lung adenocarcinoma, STAD–stomach adenocarcinoma, LIHC–liver hepatocellular carcinoma, COAD–colon adenocarcinoma, READ–rectal adenocarcinoma, UCEC–uterine corpus endometrial carcinoma, CESC–cervix squamous cell carcinoma and endocervical adenocarcinoma, PRAD–prostate adenocarcinoma. **B** Confusion matrix with the results of the anomaly detection layer for the negative control samples (*n* = 124). Figure S5. Factors influencing the classifier results. Correlation between the probability score of the correct class and the **A** CD3, **B** CD20 immune infiltrate, **C** Ki-67 proliferation rate, and **D** tumor purity. Comparison of the probability score of the correct class between **E** PAAD metastasis locations, **F** morphology, **G** tumor grade, **H** p53 expression pattern, **I** SMAD4 expression pattern, **J** GATA6 level, **K** CK7/20 expression profile, **L** ANXA1/10 score, and **M** IHC score. **N** Representative H&E staining and IHC characterization of a primary PAAD and matched PAAD met.^PC^ organoid. **O** Copy number plot for primary PAAD organoid and for PAAD met.^PC^ organoid model. The plots show the chromosomal alterations at the respective CpG sites, deletions are below and gains above the baseline located at 0. Figure S6. DNA methylation-associated organotropism of pancreatic ductal adenocarcinoma metastases. **A** Summary copy number plot for PAAD met.^Liv.^ (*n* = 21). **B** Summary copy number plot for primary PAAD (*n* = 16). **C** Summary copy number plot for PAAD met.^PC^ (*n* = 11). The plots show the frequency of chromosomal alterations at the respective CpG sites, deletions are below and gains above the baseline located at 0. Additionally, 14 genes with known roles in PAAD are highlighted. Figure S7. Pathway analysis for differentially methylated probes of enhancers and promoters using methylGSA. **A** Pathways reaching an overlap of over 10% between discovered genes and pathway genes for comparing differentially methylated probes between PAAD met.^Liv.^ and PAAD met.^PC^ **B** Pathways reaching an overlap of over 10% between discovered genes and pathway genes for comparing differentially methylated probes between primary PAAD and PAAD met.^Liv.^. Purple marks pathways also discovered by using Enrichr. Table S1. Characteristics of the internal set of brain metastases. Table S2. Tumor purity estimated by the pathologist and number of slides used for the DNA extraction. Table S4. Patient characteristics of the positive control samples. Table S5. Histological and immunohistochemical characteristics of the positive control samples. Table S6. Histological and immunohistochemical characteristics of the PAAD met.^Liv.^ samples.

Supplementary Material 2: Table S3. Detailed overview of the binomial filter scores and neural network classification scores of all included samples (Excel Table).

Supplementary Material 3: Table S7. List of genes associated to differentially methylated CpGs mapping to promoters and enhancers in pairwise comparison: PAAD primary versus PAAD met.^Liv.^, PAAD met.^Liv.^ versus PAAD met.^PC^, and PAAD primary versus PAAD met.^PC^, respectively (Excel Table).

Supplementary Material 4: Table S8. Top 10 pathways of gene sets of differentially methylated CpGs associated with promoters and enhancers in pairwise comparison: PAAD primary versus PAAD met.^Liv.^, PAAD met.^Liv.^ versus PAAD met.^PC^, and PAAD primary versus PAAD met.^PC^, respectively. Pathways associated with epithelial-mesenchymal transition (EMT) are marked in light red (Excel Table).

## Data Availability

The in-house clinical dataset analyzed in this study is available from the Gene Expression Omnibus (GEO) repository under the following accession numbers: GSE252130 and GSE217384.

## Abbreviations

ANXA1: Annexin 1
ANXA10: Annexin 10
BRCA: Breast invasive carcinoma
CNA: Copy number alteration
CESC: Cervical squamous cell carcinoma and endocervical adenocarcinoma
CK: Cytokeratin
CK20: Cytokeratin 20
CK7: Cytokeratin 7
COAD: Colon adenocarcinoma
CUP: Cancers of unknown primary
DMA: Differentially methylated analysis
DMP: Differentially methylated probes
EMT: Epithelial-mesenchymal transition
ESCA: Esophageal carcinoma
FFPE: Formalin fixed paraffin embedded
GI: Tract gastrointestinal tract
iCCA: Intrahepatic cholangiocarcinoma
IHC: Immunohistochemistry
IRS: Immunoreactive score
LIHC: Liver hepatocellular carcinoma
LN: Lymph node
LUAD: Lung adenocarcinoma
PAAD: Pancreatic ductal adenocarcinoma
PC: Peritoneal carcinomatosis
PRAD: Prostate adenocarcinoma
READ: Rectum adenocarcinoma
STAD: Stomach adenocarcinoma
t-SNE: T-distributed stochastic neighbor embedding
TMA: Tissue microarrays
TME: Tumor microenvironment
UCEC: Uterine corpus endometrial carcinoma
