# Supplementary material for: DNA methylation classifier to diagnose pancreatic ductal adenocarcinoma metastases from different anatomical sites
Source: Clin Epigenetics. 2024 Nov 10;16:156. doi: 10.1186/s13148-024-01768-x (PMC11550539; doi:10.1186/s13148-024-01768-x)
Supplement: Supplementary file 1 — Supplementary Material 1: Figure S1. Upgrading the classifier. A Confusion matrix with the results of the anomaly detection layer for the biological validation samples (n = 3579). B Comparison of the probability score of the correct class between fresh frozen and FFPE tissue in the validation cohort. C Confusion matrix with the results of the anomaly detection layer for the technical validation samples (n = 15). D Confusion matrix with the classifier results of the technical validation samples - EPICv2 (n = 15). Figure S2. Characterization of the positive control samples. A Examples of H&E and IHC staining of peritoneal carcinomatosis from PAAD, B PAAD lung metastasis, C PAAD lymph node metastasis, and D PAAD liver metastasis. E Overview of the patient characteristics. Figure S3. t-SNE analysis of the reference and positive control samples. The two-dimensional representation of the reference cohort and positive control samples (n = 415) using the t-SNE method based on DNA methylation profiles. The color code of the samples represents: A the origin of the study set, B material type, C array type, D metastases origin, E IHC profile suggestive for, and F tumor purity. Figure S4. Off label use of the classifier. A The two-dimensional plot representation using the t-SNE method, based on the DNA methylation profiles of the positive control group (n = 16) together with reference samples (n = 399) and anomaly detection samples (10 different carcinomas, n = 787). BRCA—breast invasive carcinoma, ESCA—esophageal carcinoma, LUAD—lung adenocarcinoma, STAD–stomach adenocarcinoma, LIHC–liver hepatocellular carcinoma, COAD–colon adenocarcinoma, READ–rectal adenocarcinoma, UCEC–uterine corpus endometrial carcinoma, CESC–cervix squamous cell carcinoma and endocervical adenocarcinoma, PRAD–prostate adenocarcinoma. B Confusion matrix with the results of the anomaly detection layer for the negative control samples (n = 124). Figure S5. Factors influencing the classifier results. Correla [file 13148_2024_1768_MOESM1_ESM.pdf]

Supplemental figures and tables

DNA methylation classifier to diagnose pancreatic ductal adenocarcinoma metastases from different anatomical sites

Teodor G. Calina et al.

Supplemental Figures S1–S7 Pages 2-12

Supplemental Tables S1-S8 Pages 13-15

Supplementary Figures

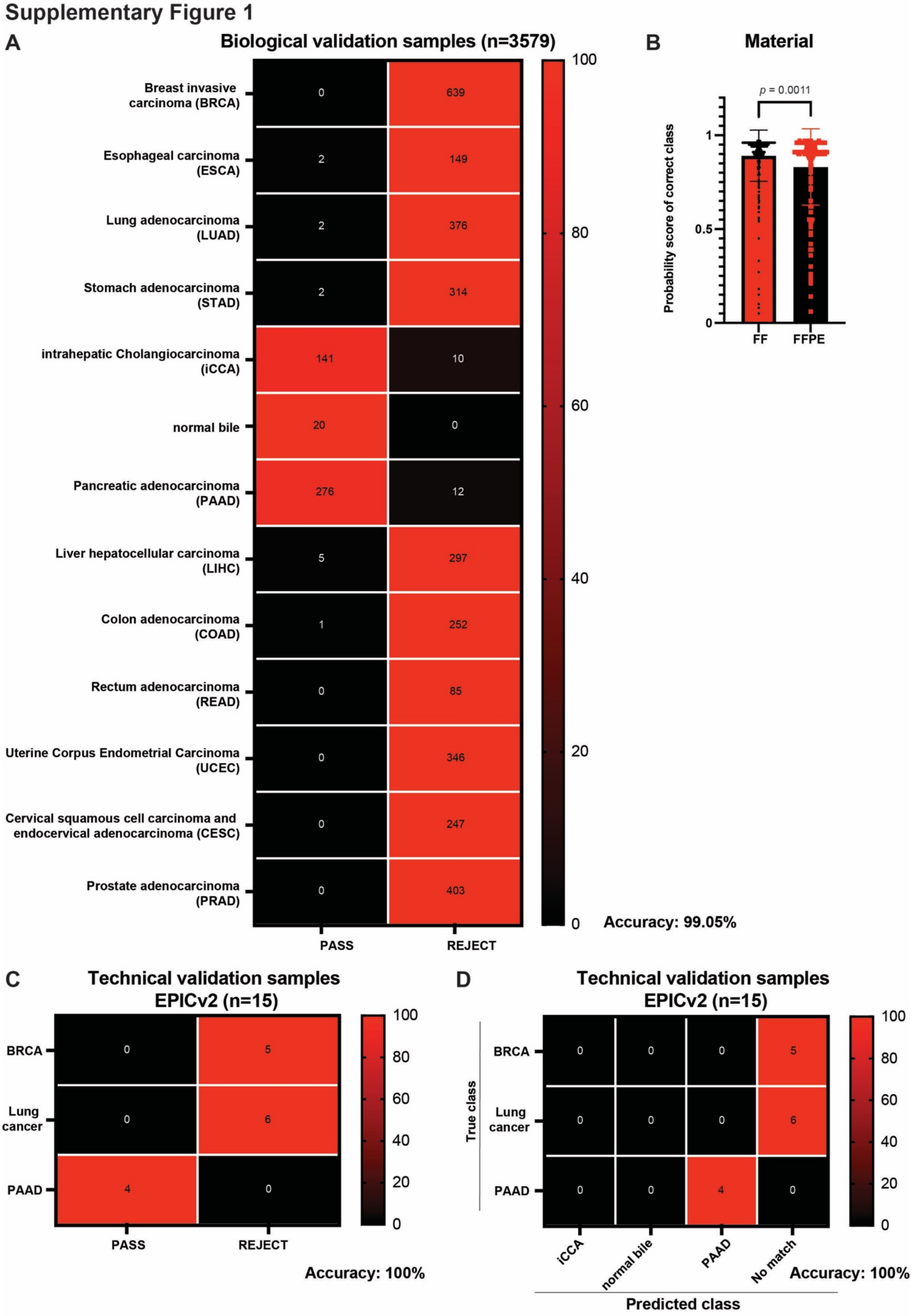

Figure S1. Upgrading the classifier. A. Confusion matrix with the results of the anomaly detection layer for the biological validation samples (n=3579). B. Comparison of the probability score of the correct class between fresh frozen and FFPE tissue in the validation cohort. C. Confusion matrix with the results of the anomaly detection layer for the technical validation samples - EPICv2 (n=15). D. Confusion matrix with the classifier results of the technical validation samples - EPICv2 (n=15).

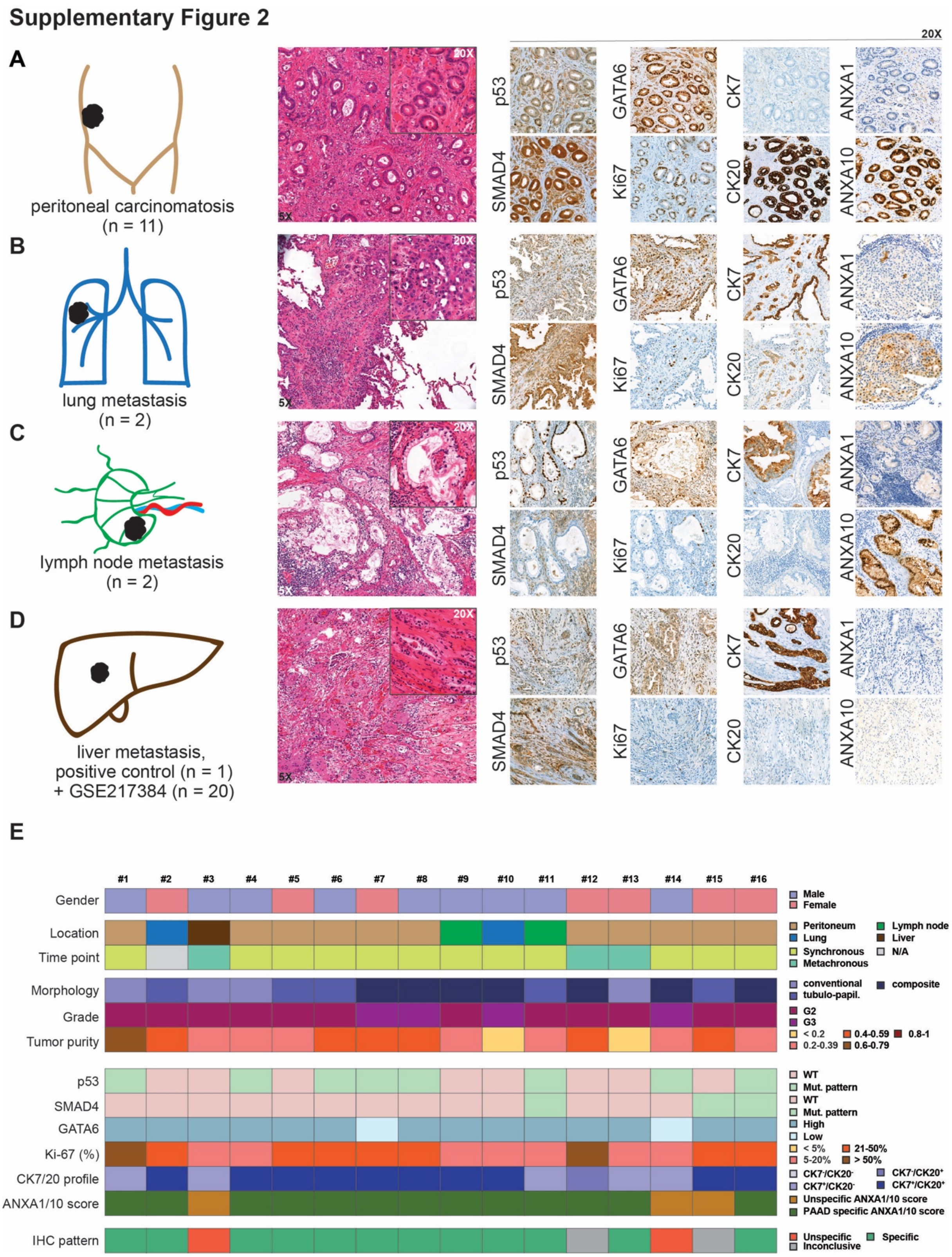

Fig. S2. Characterization of the positive control samples. A. Examples of H&E and IHC staining of peritoneal carcinomatosis from PAAD, **B.** PAAD lung metastasis, **C.** PAAD lymph node metastasis, and **D.** PAAD liver metastasis. **E.** Overview of the patient characteristics.

Supplementary Figure 3

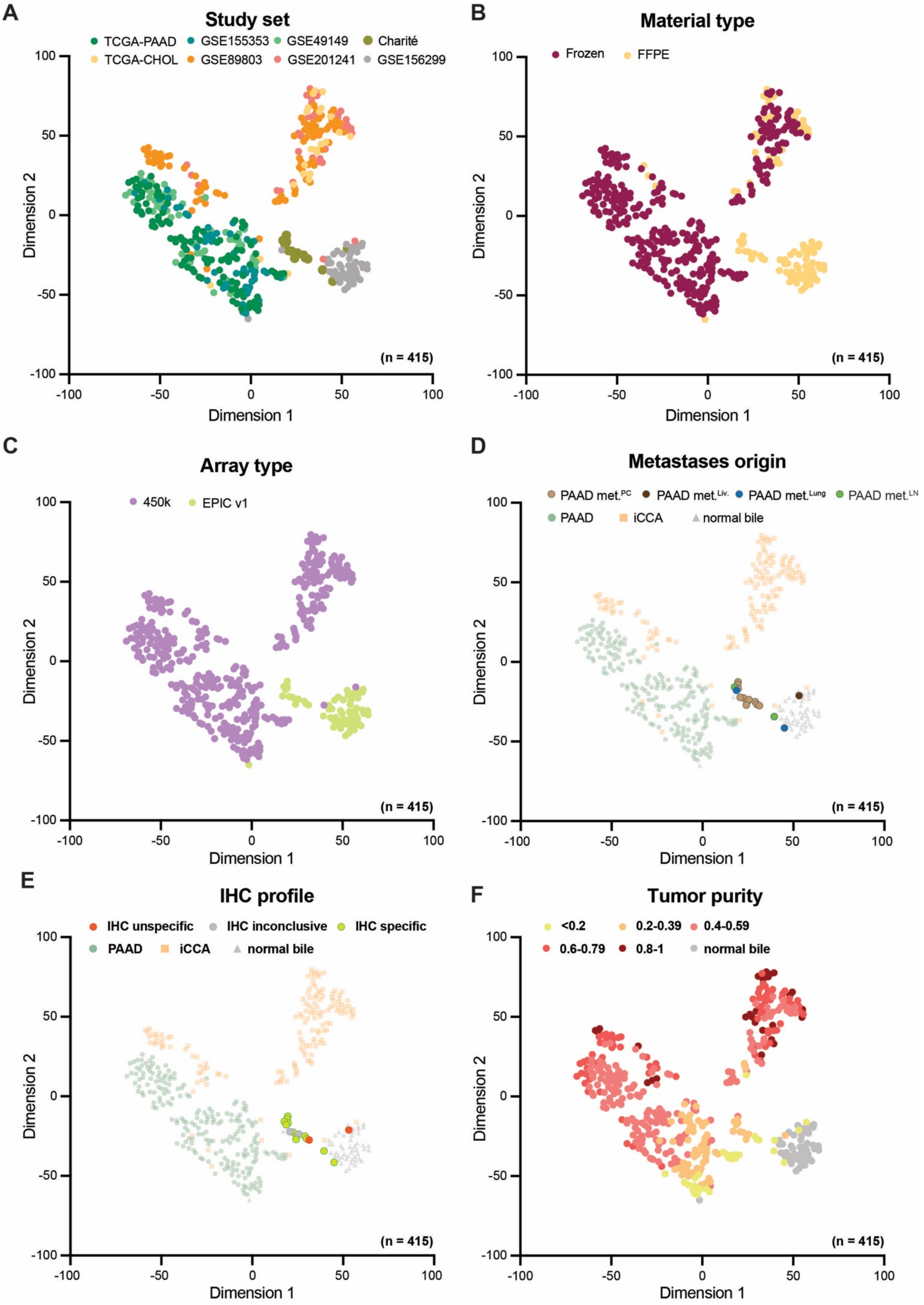

Fig. S3. t-SNE analysis of the reference and positive control samples. The two-dimensional representation of the reference cohort and positive control samples (n=415) using the t-SNE method based on DNA methylation profiles. The color code of the samples represents: **A.** the origin of the study set, **B.** material type, **C.** array type, **D.** metastases origin, **E.** IHC profile suggestive for, and **F.** tumor purity.

Supplementary Figure 4

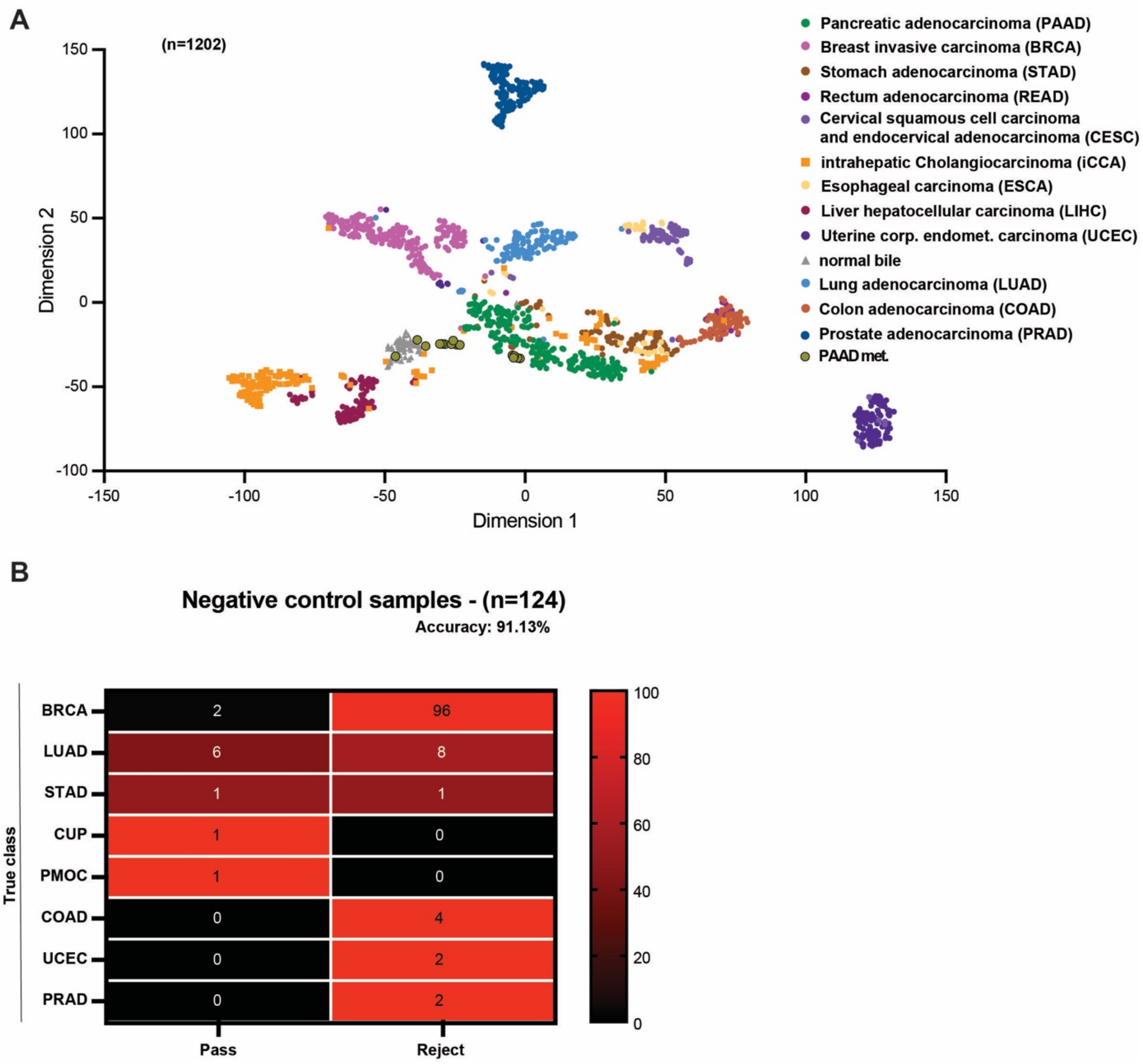

Fig. S4. **Off label use of the classifier. A**. The two-dimensional plot representation using the t-SNE method, based on the DNA methylation profiles of the positive control group (n=16) together with reference samples (n=399) and anomaly detection samples (10 different carcinomas, n=787). BRCA – breast invasive carcinoma, ESCA – esophageal carcinoma, LUAD – lung adenocarcinoma, STAD – stomach adenocarcinoma, LIHC – liver hepatocellular carcinoma, COAD – colon adenocarcinoma, READ – rectal adenocarcinoma, UCEC – uterine corpus endometrial carcinoma, CESC – cervix squamous cell carcinoma and endocervical adenocarcinoma, PRAD – prostate adenocarcinoma. **B**. Confusion matrix with the results of the anomaly detection layer for the negative control samples (n=124).

Supplementary Figure 5

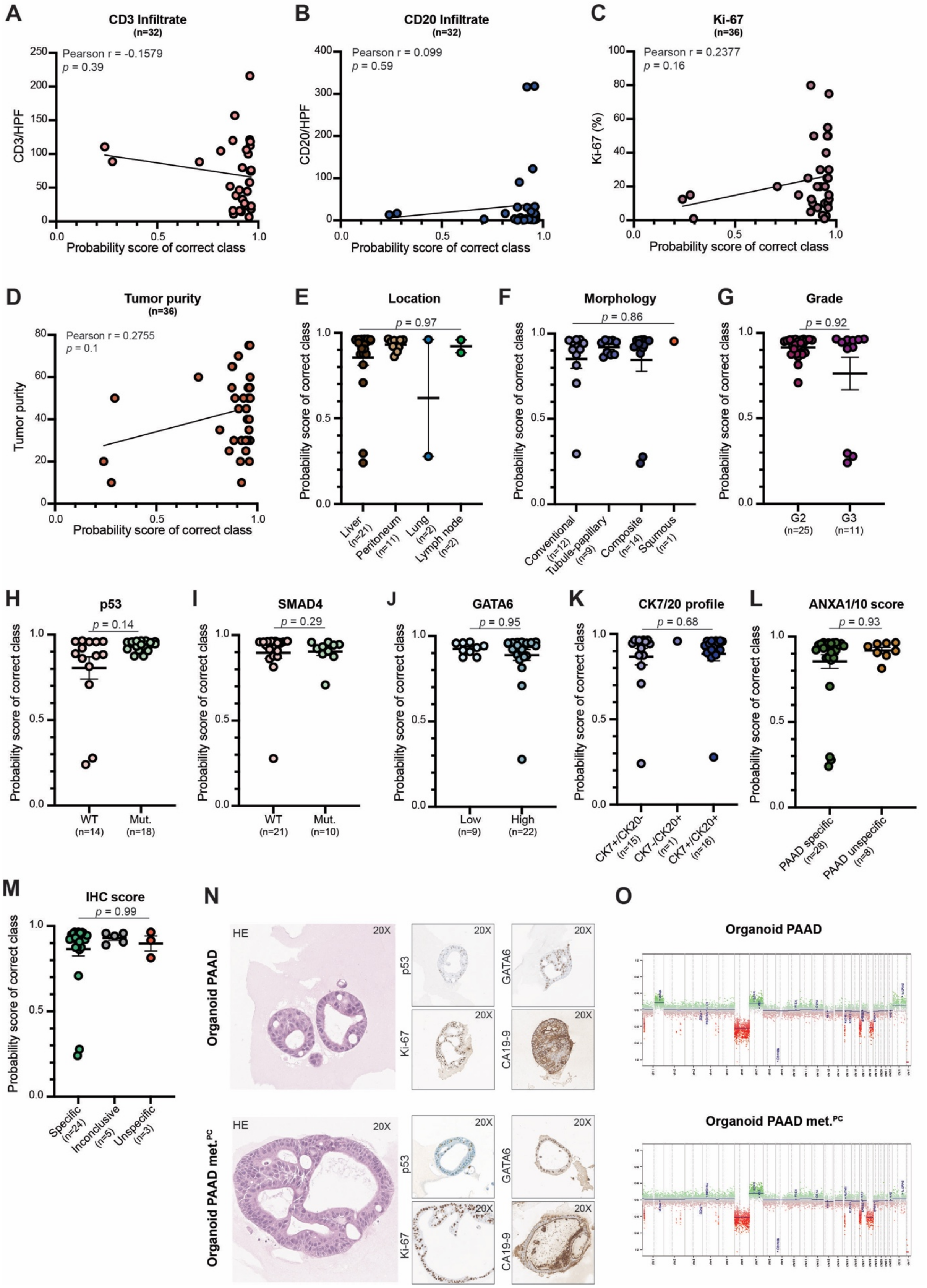

Fig. S5. Factors influencing the classifier results. Correlation between the probability score of the correct class and the **A.** CD3, **B.** CD20 immune infiltrate, **C.** Ki-67 proliferation rate, and **D.** tumor purity. Comparison of the probability score of the correct class between **E.** PAAD metastasis locations, **F.** morphology, **G**. tumor grade, **H.** p53 expression pattern, **I**. SMAD4 expression pattern, **J**. GATA6 level, **K.** CK7/20 expression profile, **L.** ANXA1/10 score, and **M.** IHC score. **N.** Representative H&E staining and IHC characterization of a primary PAAD and matched PAAD met.^{PC} organoid. **O.** Copy number plot for primary PAAD organoid and for PAAD met.^{PC} organoid model. The plots show the chromosomal alterations at the respective CpG sites, deletions are below and gains above the baseline located at 0.

Supplementary Figure 6

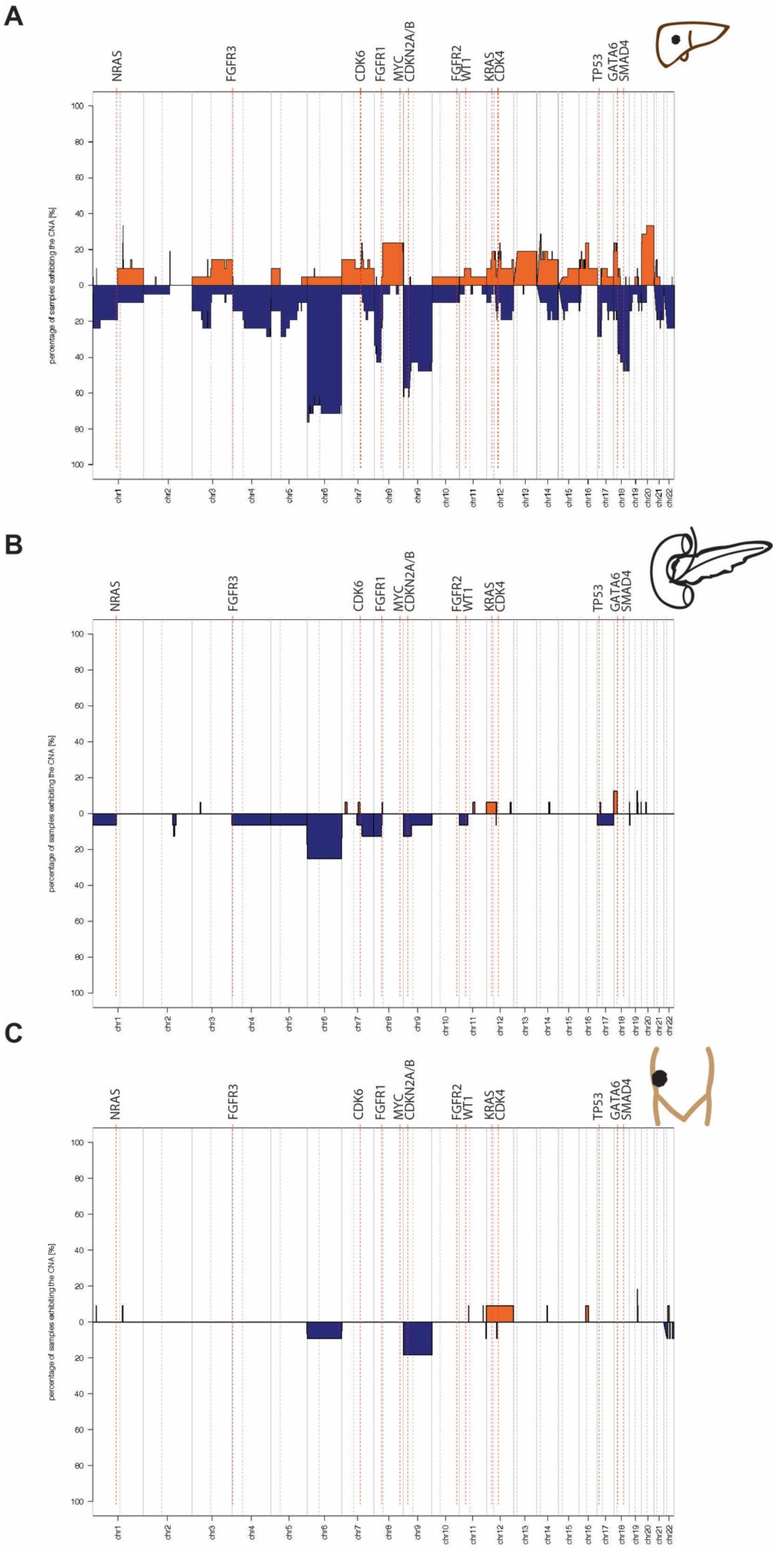

Fig. S6. DNA methylation-associated organotropism of pancreatic ductal adenocarcinoma metastases. A. Summary copy number plot for PAAD met.^{Liv} (n=21). **B**. Summary copy number plot for primary PAAD (n=16). **C.** Summary copy number plot for PAAD met.^{PC} (n=11). The plots show the frequency of chromosomal alterations at the respective CpG sites, deletions are below and gains above the baseline located at 0. Additionally, 14 genes with known roles in PAAD are highlighted.

Supplementary Figure 7

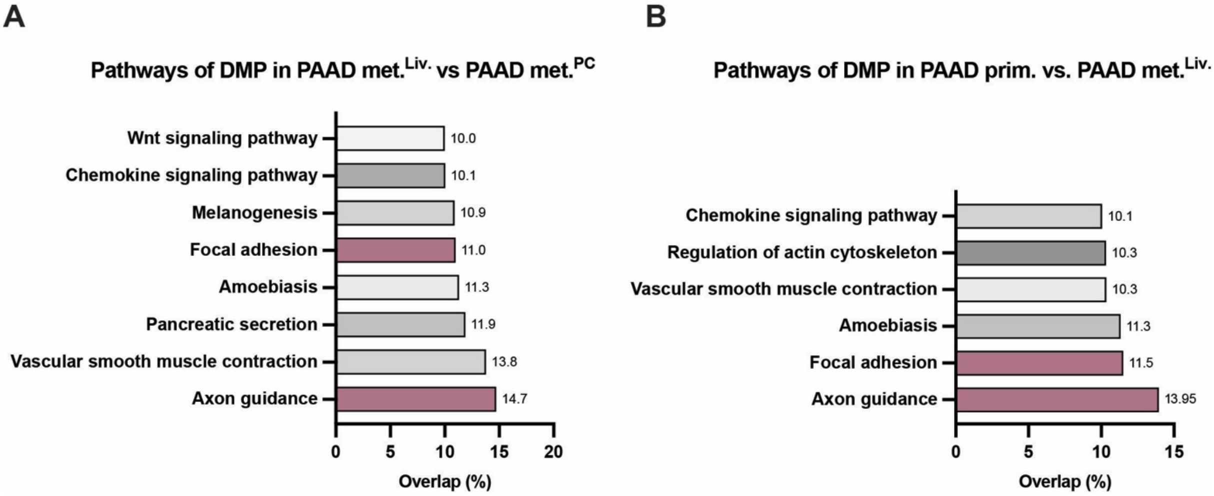

Fig. S7. Pathway analysis for differentially methylated probes of enhancers and promoters using methylGSA. A. Pathways reaching an overlap of over 10% between discovered genes and pathway genes for comparing differentially methylated probes between PAAD met.$^{Liv.}$ and PAAD met.^{PC} **B**. Pathways reaching an overlap of over 10% between discovered genes and pathway genes for comparing differentially methylated probes between primary PAAD and PAAD met.$^{Liv.}$. Purple marks pathways also discovered by using Enrichr.

Supplemental Tables

Supplemental Table S1. Characteristics of the internal set of brain metastases.

Chip ID	Location	Suggested diagnosis by clinical history, imaging, histology IHC, and molecular diagnosis
205751520032_R05C01	Brain	Non-small cell lung cancer, adenocarcinoma
207695720028_R01C01	Brain	Non-small cell lung cancer, adenocarcinoma
203810640132_R06C01	Brain	Endometroid endometrium carcinoma of the uterus
205832320093_R08C01	Brain	Primary mucinous ovarian cancer
205041760083_R05C01	Brain	Adenocarcinoma CUP
203808720090_R02C01	Brain	Non-small cell lung cancer, adenocarcinoma
203810690124_R03C01	Brain	Non-small cell lung cancer, adenocarcinoma
203821710048_R06C01	Brain	Non-small cell lung cancer, adenocarcinoma
203013210156_R01C01	Brain	Non-small cell lung cancer, adenocarcinoma
201465930022_R06C01	Brain	Gastric adenocarcinoma
205041750127_R02C01	Brain	Non-small cell lung cancer, adenocarcinoma
205096370066_R01C01	Brain	Breast cancer (triple negative)
205751530142_R02C01	Brain	Gastric adenocarcinoma
205689140042_R03C01	Vertebral column	Prostate carcinoma
207419460035_R04C01	Cerebellum	Endometroid endometrium carcinoma of the uterus

Supplemental Table S2. Tumor purity estimated by the pathologist and number of slides used for the DNA extraction.

Chip ID	Tumor purity	Slides
206687210091_R01C01	60%	20X
206687210091_R02C01	55%	10X
206687210091_R03C01	35%	10X
206687210091_R04C01	25%	10X
206687210091_R05C01	25%	15X
206687210091_R06C01	40%	15X
206687210091_R07C01	40%	15X
206687210091_R08C01	50%	15X
207179230128_R01C01	30%	20X
207179230128_R02C01	10%	20X
207179230128_R03C01	35%	10X
207179230128_R04C01	50%	7X
207179230128_R05C01	10%	20X
207179230128_R06C01	20%	15X
207179230128_R07C01	50%	15X
207179230128_R08C01	30%	20X

Supplemental Table S3. Detailed overview of the binomial filter scores and neural network classification scores of all included samples (Excel Table).

Supplemental Table S4. Patient characteristics of the positive control samples.

ID	Scan ID	Age	Sex	Location	Clinical history	Primary tumour
#1	206687210091_R01C01	50	M	peritoneal carcinomatosis	Imagistic suspect infiltrative mass in the pancreas head	Synchronous
#2	206687210091_R02C01	73	W	lung metastasis	Imagistic suspect mass in the pancreas	N/A
#3	206687210091_R03C01	51	M	liver metastasis (positive control)	2 years prior, resected pancreas tail with confirmed PAAD.	Metachronous
#4	206687210091_R04C01	70	M	peritoneal carcinomatosis (mesenterium)	Simultaneous resection of pancreas tail with confirmed PAAD	Synchronous
#5	206687210091_R05C01	74	W	peritoneal carcinomatosis	Clinical suspicion of pancreas carcinoma	Synchronous
#6	206687210091_R06C01	77	M	peritoneal carcinomatosis	Clinical suspicion of pancreas carcinoma	Synchronous
#7	206687210091_R07C01	69	W	peritoneal carcinomatosis	Imagistic suspect infiltrative mass in the pancreas with suspected liver and suprarenal metastases	Synchronous
#8	206687210091_R08C01	85	M	peritoneal carcinomatosis (omentum)	Imagistic suspect infiltrative mass in the pancreas with suspected liver metastases	Synchronous
#9	207179230128_R01C01	60	M	lymph node metastasis with extra-nodal extension (truncus coeliacus, submitted for frozen section)	Histologic confirmed and simultaneously resected PAAD with lymph node metastases	Synchronous
#10	207179230128_R02C01	60	M	lung metastasis	Imagistic suspect infiltrative mass in the pancreas	Synchronous
#11	207179230128_R03C01	61	M	lymph node metastasis (truncus coeliacus)	Histologic confirmed and simultaneously resected PAAD with lymph node metastases	Synchronous
#12	207179230128_R04C01	63	W	peritoneal carcinomatosis (with abdominal muscle invasion)	2 years prior resected pancreas head with confirmed PAAD	Metachronous
#13	207179230128_R05C01	53	W	peritoneal carcinomatosis	1 years prior resected pancreas head with confirmed PAAD	Metachronous
#14	207179230128_R06C01	56	M	peritoneal carcinomatosis (adipose tissue of the liver hilum)	Pancreas head resection with simultaneous metastasis in liver hilum	Synchronous
#15	207179230128_R07C01	74	W	peritoneal carcinomatosis	Imagistic suspect infiltrative mass in the pancreas head	Synchronous
#16	207179230128_R08C01	62	W	peritoneal carcinomatosis	Imagistic suspect infiltrative mass in the pancreas head-body, diagnostic laparoscopy	Synchronous

PAAD – pancreas adenocarcinoma.

Supplemental Table S5. Histological and immunohistochemical characteristics of the positive control samples.

ID	Morphology	Grade	p53	SMAD4	GATA6	Ki67	CK7/20 Pattern	ANXA1	ANXA10	CD3	CD20
#1	Conventional	G2	Mut	WT	high (4)	75%	CK7+/CK20-	4	12	74.4	12.2
#2	Tubulo-papillary	G2	WT	WT	high (4)	25%	CK7+/CK20+	0	12	112.4	32
#3	Conventional	G2	WT	WT	high (4)	15%	CK7+/CK20-	0	0	104.5	16.4
#4	Conventional	G2	Mut	WT	high (4)	10%	CK7+/CK20+	0	2	23.4	1.4
#5	Tubulo-papillary	G2	WT	WT	high (4)	25%	CK7+/CK20+	3	12	52	3.6
#6	Tubule-papillary	G2	Mut	WT	high (4)	40%	CK7+/CK20+	0	12	100	122.6
#7	Composite	G3	Mut	WT	low (2)	50%	CK7+/CK20+	0	12	75.2	32.6
#8	Composite	G3	Mut	WT	high (4)	50%	CK7+/CK20+	8	12	57.8	6
#9	Composite	G2	WT	WT	high (4)	10%	CK7+/CK20+	0	4	157	90,6
#10	Composite	G3	WT	WT	high (4)	15%	CK7+/CK20+	0	8	88.8	16.8
#11	Tubulo-papillary	G2	Mut	Mut	high (4)	10%	CK7+/CK20-	4	12	215.8	318.2
#12	Composite	G2	WT	WT	high (4)	55%	CK7-/CK20+	0	12	57.8	1.6
#13	Conventional	G1	WT	WT	high (4)	20%	CK7+/CK20-	0	12	32.6	30.8
#14	Composite	G3	Mut	WT	low (2)	20%	CK7+/CK20-	0	0	14.4	3.4
#15	Tubulo-papillary	G2	WT	Mut	high (3)	50%	CK7+/CK20+	0	0	38.6	6.6
#16	Composite	G2	Mut	Mut	high (4)	30%	CK7+/CK20+	0	4	79.8	316.6

Supplemental Table S6. Histological and immunohistochemical characteristics of the PAAD met.$^{Liv.}$ samples.

Scan Nr.	Tumor purity	Morphology	Grading	p53	SMAD4	GATA6	Ki67	CK7/20 Pattern	ANXA1	ANXA10	CD3	CD20
205814850073_R01C01	55%	Composite	G3	Mut	WT	low (2)	30%	CK7+/CK20-	0	0	76	11.4
205814850073_R02C01	30%	Composite	G2	N/A	N/A	N/A	15%	N/A	3	12	18	3.5
205814850073_R03C01	30%	Conventional	G2	N/A	N/A	N/A	2.5%	N/A	3	4	N/A	N/A
205814850073_R04C01	75%	Composite	G2	WT	Mut	low (2)	55%	CK7+/CK20+	0	0	121.2	2.4
205828600120_R06C01	50%	Conventional	G3	WT	WT	high (3)	12.5%	CK7+/CK20-	3	12	22.8	3
205828600120_R07C01	65%	Tubulo-papillary	G2	WT	WT	high (4)	12.5%	CK7+/CK20+	0	12	10.8	1.4
205828600120_R08C01	55%	Tubulo-papillary	G2	Mut	Mut.	low (2)	5%	CK7+/CK20-	0	8	120	31.2
205854140048_R08C01	50%	Conventional	G3	N/A	N/A	N/A	1%	N/A	8	6	N/A	N/A
205814880008_R06C01	20%	Composite	G3	N/A	N/A	N/A	7.5%	N/A	3	0	N/A	N/A
206905700096_R01C01	75%	Tubulo-papillary	G2	Mut	WT	high (3)	15%	CK7+/CK20-	0	12	N/A	N/A
206905700096_R02C01	50%	Composite	G2	Mut	Mut	Low (2)	3%	CK7+/CK20+	0	6	26.8	2.4
206905700096_R03C01	30%	Composite	G2	Mut	Mut	low (2)	1%	CK7+/CK20-	0	0	106.2	21.3
206905700096_R04C01	45%	Conventional	G3	Mut	WT	low (2)	7.5%	CK7+/CK20+	4	8	15.8	0.8
206905700096_R05C01	20%	Composite	G3	WT	N/A	N/A	12.5%	CK7+/CK20-	2	9	110.5	13.4
206905700096_R06C01	55%	Conventional	G2	WT	WT	high (4)	25%	CK7+/CK20+	3	12	118.4	16.4
206905700096_R07C01	70%	Conventional	G2	Mut	Mut.	high (3)	20%	CK7+/CK20+	0	0	46.4	2.4
206905700097_R01C01	60%	Conventional	G2	WT	Mut.	high (4)	20%	CK7+/CK20-	3	12	88.4	3
206905700097_R02C01	55%	Squamous	G2	Mut	WT	high (3)	25%	CK7+/CK20-	4	12	6.4	1.2
206905700097_R03C01	45%	Tubulo-papillary	G3	Mut	Mut.	high (4)	20%	CK7+/CK20-	0	8	44.6	3.8
206905700097_R04C01	65%	Conventional	G2	Mut	WT	low (2)	80%	CK7+/CK20-	0	8	16	0.4

Supplemental Table S7. List of genes associated to differentially methylated CpGs mapping to promoters and enhancers in pairwise comparison: primary PAAD versus PAAD met.$^{Liv.}$, PAAD met.$^{Liv.}$ versus PAAD met.^{PC}, and primary PAAD versus PAAD met.^{PC}, respectively (Excel Table).

Supplemental Table S8. Top 10 pathways of gene sets of differentially methylated CpGs associated to promoters and enhancers in pairwise comparison: primary PAAD versus PAAD met.$^{Liv.}$, PAAD met.$^{Liv.}$ versus PAAD met.^{PC}, and primary PAAD versus PAAD met.^{PC}, respectively. In light red are marked pathways associated to epithelial-mesenchymal transition (EMT) (Excel Table).
